# Deletion of *Mettl3* in mesenchymal stem cells promotes acute myeloid leukemia resistance to chemotherapy

**DOI:** 10.1038/s41419-023-06325-7

**Published:** 2023-12-05

**Authors:** Xinai Liao, Danni Cai, Jingru Liu, Haoran Hu, Ruolan You, Zhipeng Pan, Shucheng Chen, Kaiming Xu, Wei Dai, Shuxia Zhang, Xinjian Lin, Huifang Huang

**Affiliations:** 1 Central Laboratory, Fujian Medical University Union Hospital, 350001 Fuzhou, Fujian China; 2 Fujian Institute of Hematology, Fujian Provincial Key Laboratory on Hematology, Fujian Medical University Union Hospital, 350001 Fuzhou, Fujian China; 3 Key Laboratory of Gastrointestinal Cancer (Fujian Medical University), Ministry of Education, 350122 Fuzhou, Fujian China

**Keywords:** Cancer, Stem cells, Diseases

## Abstract

Acute myeloid leukemia (AML) cell survival and chemoresistance are influenced by the existence of bone marrow mesenchymal stem cells (BMMSCs); however, the pathways by which BMMSCs contribute to these processes remain unclear. We earlier revealed that methyltransferase-like 3 (METTL3) expression is significantly reduced in AML BMMSCs and that METTL3 mediates BMMSC adipogenesis to promote chemoresistance in human AML cell lines in vitro. In this investigation, we evaluated the METTL3 function in vivo. Mice exhibiting a conditional removal of *Mettl3* in BMMSCs were developed by mating *Prrx1-Cre*^*ERT2*^*;Mettl3*^*fl/+*^ mice with *Mettl3*^*fl/fl*^ mice using the CRISPR-Cas9 system. The *Mettl3* deletion increased bone marrow adiposity, enhanced disease progression in the transplantation-induced MLL-AF9 AML mouse model, and chemoresistance to cytarabine. The removal of *Mettl3* in BMMSCs resulted in a significant increase in BMMSC adipogenesis. This effect was attributed to the downregulation of AKT1 expression, an AKT serine/threonine kinase 1, in an m^6^A-dependent manner. The development of chemoresistance in AML is linked to the promoted adipogenesis of BMMSCs. We conclude that METTL3 expression in BMMSCs has a critical function in limiting AML progression and chemoresistance, providing a basis for the progression of therapeutic approaches for AML.

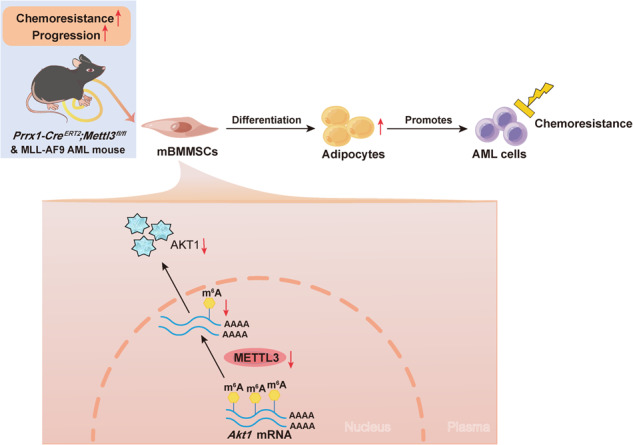

## Introduction

Acute myeloid leukemia (AML) is defined by immature myeloid blast accumulation in the peripheral blood (PB), bone marrow (BM), and other organs [1, 2]. Despite advances in therapy, some patients experience poor outcomes, with frequent resistance or relapse [3, 4]. Therefore, novel therapeutic strategies are urgently needed to address refractory or relapsed AML. Increased leukemic cell adhesion, production of growth factors and cytokines, and enhanced immunosuppression are all aspects of the BM microenvironment (BMM) that are related to leukemogenesis and chemoresistance [5, 6]. Thus, the BMM is a promising treatment target for managing AML.

Evidence indicates that mesenchymal stem cells (MSCs) have the ability to enhance AML cell survival, facilitate BM engraftment, and promote resistance to therapy [7, 8]. MSCs demonstrate robust self-renewal potential and can differentiate into three primary mesodermal lineages, namely adipocytes, chondrocytes, and osteoblasts [9]. Adipocytes are active BM cells that accumulate free fatty acids and release adipokines, which support tumor development and treatment resistance [10–12]. In addition, adipocytes promote the high-energy lipid transfer and mitochondrial metabolism essential for tumor cell survival and growth [13]. The co-cultivation of leukemia cells with adipocytes causes a significant decrease in the effectiveness of chemotherapy against leukemia. Additionally, a correlation exists between the bone marrow mesenchymal stem cells (BMMSCs) adipogenic differentiation and a rise in leukemic engraftment in mice [14, 15]. Increased BM adipocyte content stimulates AML cell growth, which is associated with a poor prognosis [16]. These outcomes indicate that increased BM adipocytes can induce leukemia progression and chemoresistance.

The most common mRNA posttranscriptional modification, N^6^-methyladenosine (m^6^A), has been shown to impact various biological processes [17, 18]. The methyltransferase compound, which comprises Wilms’ tumor 1-associating protein (WTAP), methyltransferase-like 14 (METTL14), and methyltransferase-like 3 (METTL3), is responsible for catalyzing m^6^A in mammals [19, 20]. AML cells exhibit a high level of expression of METTL3, which has been observed to expedite the progression of leukemia in murine models [21]. A recent study has highlighted the significant role of METTL3 in AML chemoresistance and suggested promising applications for METTL3-mediated m^6^A modification in the management of refractory/relapsed AML [22]. In addition, METTL3 suppresses adipogenesis in porcine BM-derived MSCs [23], and the knockout of *Mettl3* in BMMSCs enhances adipogenesis in mice [24]. Our prior investigation demonstrated that METTL3 expression is significantly decreased in AML BMMSCs in contrast to their healthy donor counterparts and that METTL3 mediates BMMSC adipogenesis to promote chemoresistance in human AML cell lines in vitro [25]. However, evidence supporting the in vivo impact of METTL3 on the adipogenic differentiation of BMMSCs, AML progression, and response to chemotherapy is still lacking.

Here, we used MSC-specific inducible *Mettl3* knockout mice *Prrx1-Cre*^*ERT2*^*;Mettl3*^*fl/fl*^ and an MLL-AF9 AML mouse model to define the influence of *Mettl3* deficiency on AML progression, response to chemotherapy in vivo, and the underlying cellular and molecular pathways.

## Materials and methods

### Generation of conditional *Mettl3* knockout mice

The CRISPR-Cas9 system produced *Mettl3*^*fl/+*^ mice with a C57BL/6J background to create a conditional knockout model. The exon2-exon4 region of the *Mettl3*-201 (ENSMUST00000022767.15) transcript was identified as the ideal knockout region depending on the construction of the *Mettl3* gene. This region contains a 799 bp coding sequence. For the *Mettl3*^*fl/+*^ mice, two single guide RNAs (sgRNA1: 5′-GGTTTATTGTATCATTTGAG-3′; sgRNA2: 5′-CCGGTGTTTACTCTGGAGTA-3′) were constructed and transcribed in vitro. Concurrently, the donor vector containing the *loxP* fragment was designed and constructed. Next, the Cas9 mRNA, sgRNAs and donor fragments were co-microinjected into fertilized eggs obtained from C57BL/6J mice. These zygotes were then transferred into the oviduct of pseudopregnant ICR females. After a gestation period of 19–21 days, F0 mice were born, and their genotypes were determined through PCR and sequencing of tail DNA. Finally, a stable line of F1 generation heterozygous mice was established by mating F0-positive mice with C57BL/6J mice. This entire process was expertly executed by GemPharmatech Co., Ltd (Nanjing, China).

The *Prrx1-Cre*^*ERT2*^ transgenic mice employed in this investigation were acquired from The Jackson Laboratory (Bar Harbor, ME, USA). *Prrx1-Cre*^*ERT2*^ and *Mettl3*^*fl/+*^mice were crossed to obtain *Prrx1-Cre*^*ERT2*^*;Mettl3*^*fl/+*^ mice. By mating *Prrx1-Cre*^*ERT2*^*;Mettl3*^*fl/+*^ mice with *Mettl3*^*fl/fl*^ mice, *Prrx1-Cre*^*ERT2*^*;Mettl3*^*fl/fl*^ mice were generated as homozygous conditional *Mettl3* knockout mice. *Cre*^*ERT2*^ recombinase was activated by tamoxifen (TAM) treatment (Cat# HY-13757A, MCE, Monmouth Junction, NJ, USA) (150 mg/kg/day, i.p. for 5 consecutive days, 20 mg/mL solution in corn oil).

Utilizing the primers specified in Supplementary Table S1, a PCR analysis of the mouse tails’ genomic DNA was performed to identify the genotype of transgenic mice. The PCR cycling conditions consisted of an initial step at 95 °C for 5 min, followed by 35 cycles of denaturation at 95 °C for 30 s, annealing at 55 °C for 30 s, and extension at 72 °C for 30 s. The amplification was concluded with a final extension step at 72 °C for 5 min. The mice were housed in a controlled environment with a 12/12-h light/dark cycle at 22–24 °C in specific pathogen-free (SPF) conditions. They were provided with unrestricted access to sterile pellet food and water. No statistical method was used to predetermine the sample size for the mouse experiment. Mice of the same age (8–10 weeks old), sex, and genotype were pooled and subsequently grouped randomly. No blinding methods were applied to the subsequent experiments, and no exclusion criteria for animals were used. All animal experimental protocols were conducted in compliance with guidelines outlined in the National Institutes of Health Guide for the Care and Use of Animals and were approved by the Institutional Animal Care and Use Committee of Fujian Medical University, China.

### Production of the MLL-AF9-induced AML syngeneic mouse model

The retroviral constructs MSCV-MLL-AF9-IRES-YFP (30 μg) along with packaging plasmid pCL-ECO (15 μg) were transfected into 293T cells of 80–90% confluence in a 10-cm cell culture plate utilizing Lipofectamine 3000 (Cat# L3000015, Invitrogen, Carlsbad, CA, USA) for virus packaging. After 48–72 h, the retroviral supernatant was gathered and utilized for transduction. E20 fetal liver Lin^−^ cells were suspended in viral supernatants (1 × 10^5^ cells/mL) containing 4 μg/mL polybrene, followed by centrifugation at 2000 rpm for 120 min. The cells were then cultured for 24 h in StemSpan (Cat# 09600, StemCell Technologies, Vancouver, BC, Canada) supplemented with 10 μg/mL heparin, 50 ng/mL mSCF (Cat# 250-03-50, Peprotech, Cranbury, NJ, USA), 10 ng/mL mIL-3 (Cat# 213-13-10, Peprotech), 10 ng/mL mIL-6 (Cat# 216-16-10, Peprotech), 100 units/mL of penicillin, and 100 µg/mL of streptomycin (Cat# 15140122, Gibco, Carlsbad, CA, USA). Subsequently, the cells were resuspended in viral supernatant to initiate a subsequent transduction cycle. A quantity of 3 × 10^5^ transducted Lin^−^ cells was resuspended in 200 μL phosphate-buffered saline (PBS) and then introduced into C57BL/6 J mice (8–10 weeks old) undergoing sublethal irradiation (6.5 Gy) through tail vein injection. YFP^+^ BM cells were obtained from primary recipient mice through fluorescence-activated cell sorting (FACS) for the second transplantation. Subsequently, 2 × 10^5^ YFP^+^ cells were transplanted into the irradiated (4.5 Gy) recipients. In the chemotherapy sensitivity experiments, we transplanted 2 × 10^5^ YFP^+^ cells into nonirradiated recipients for sufficient reaction time.

### Cell culture

The OP9 mouse stromal cell line and three human AML cell lines (HL-60, U-937, and THP-1) were acquired from the Cell Bank of Type Culture Collection of the Chinese Academy of Sciences in Shanghai, China. All the cell lines have been authenticated by short tandem repeat (STR) profiling. The OP9 cells were preserved in Alpha Modification Minimum Essential Medium (α-MEM; HyClone, Logan, UT, USA) that was treated with 10% fetal bovine serum (FBS; Gibco) and 100 units/mL penicillin and 100 µg/mL of streptomycin (Gibco). The AML cells were cultivated in RPMI1640 medium (RPMI1640; HyClone) and treated with 10% FBS (Gibco) and 100 units/mL penicillin and 100 µg/mL of streptomycin (Gibco). The cells were cultured under controlled temperature and humidity conditions in a CO_2_ incubator with 5% CO_2_ at 37 °C.

Primary BMMSCs were obtained from compact bones, as described previously [26]. Briefly, the BM cavities underwent a minimum of three washes to remove the hematopoietic cells. Compact bones were divided into 1–3-mm^3^ segments and digested with collagenase II (Cat# 9001-12-1, Solarbio, Beijing, China). Liberated cells were washed out of the bone into solution and cultivated in α-MEM treated with 10% FBS (Gibco) and 100 units/mL penicillin and 100 µg/mL of streptomycin (Gibco).

### Adipogenic and osteogenic differentiation assays

OP9 cells and primary BMMSCs were cultivated as a monolayer and then subjected to incubation in the adipogenesis induction medium (Cat# GUXMX-90031, Cyagen Biosciences Inc., Santa Clara, CA, USA) for 2 weeks, for adipogenic differentiation. Oil Red O (ORO) staining was incubated with ORO solution (Cat# OILR-10001, Cyagen Biosciences Inc.) for 30 min to visualize lipid droplets. The incorporated staining was then dissolved in isopropanol at room temperature and transferred to a 96-well plate for quantitative analysis. The absorbance at 500 nm was measured by a microplate reader (SpectraMax i3x, Molecular Devices, San Jose, CA, USA). The cells underwent a 3-week differentiation process into osteoblasts while being cultivated in high-glucose Dulbecco’s Modified Eagle Medium (HG-DMEM; Hyclone) treated with 50 μmol/L ascorbic acid, 10% FBS, 10^−7^ mol/L dexamethasone, and 10 mmol/L β-glycerol phosphate (Sigma-Aldrich, St. Louis, MO, USA). The cells were subjected to staining with 2% Alizarin Red Solution (ARS; Cat# G1450, Solarbio) for 10 min after 21 days of differentiation. The incorporated staining was then dissolved in 10% chlorocetylpyridine at room temperature and transferred to a 96-well plate. The absorbance at 562 nm was measured by a microplate reader.

### Co-culture and chemoresistance assays

OP9 cells or primary BMMSCs were seeded at a density of 1.0 × 10^5^ cells per well in a six-well plate. The next day, the cells were subjected to a 2-week incubation in adipogenesis induction medium to induce adipogenic differentiation. Subsequently, differentiated adipocytes were co-cultured with various AML cells, including mouse MLL-AF9 AML cells and human cell lines U-937, HL-60, and THP-1. Each of these AML cells was added at 5.0 × 10^5^ per well and the co-cultures were maintained in leukemia medium. AML cells’ sensitivity to chemotherapy was detected using the Cell Counting Kit-8 (CCK-8; Cat# CK04-11, Dojindo Laboratories, Do Jindo Laboratories, Kumamoto, Japan), as previously described [27]. Briefly, AML cells were placed onto 96-well plates in a growth medium. Mouse MLL-AF9 AML cells were exposed to 0.5 μM cytarabine (Ara-C; Cytosar, Foshan, Guangdong, China), 50 ng/mL daunorubicin (DNR; Shandong New Era Pharmaceutical Co. Ltd., Linyi, Shandong, China), or PBS (vehicle control). After incubation for 24 h, CCK-8 solution was added to each well, and the plates were incubated in darkness at 37 °C for an additional 4 h. The absorbance was measured using a microplate reader. Similarly, human AML cells were treated with 10 μM Ara-C or 150 ng/mL DNR. The percentage of cell growth inhibition rate was computed using the formula: (control group – experimental group)/(control group – blank group) ×100%.

### Enzyme-linked immunosorbent assay (ELISA)

The bone marrow was harvested from both lower extremities of the mice, flushed with a defined volume of PBS containing 2% FBS, and then centrifuged for 10 min at 1500 rpm. In the supernatant, based on the manufacturer’s directions, the levels of adipokines, like resistin, leptin, and growth hormone, were quantified employing the kits of Mouse Resistin ELISA (Cat# CSB-E06886m, CUSABIO, Wuhan, Hubei, China), Mouse Leptin ELISA (Cat# CSB-E04650m, CUSABIO), and Mouse Growth Hormone ELISA (Cat# CSB-E07343m, CUSABIO), respectively.

### Quantitative real-time PCR (qPCR)

TRIzol (Invitrogen) was employed depending on the manufacturer’s directions to obtain total RNA extraction from cells. cDNA was created using 5×All-in-One RT MasterMix with AccuRT (Cat# G592, Applied Biological Materials, Vancouver, Canada), and levels of mRNA were detected employing Eva Green 2×qPCR MasterMix-Low ROX (Cat# G891, Applied Biological Materials). Supplementary Table S2 illustrates the primer sequences. The 2^−ΔΔCt^ technique was used for data analysis, with Glyceraldehyde 3-phosphate dehydrogenase (*Gapdh*) functioning as the internal control.

### Virus production and transduction of cells

The construction of a wild-type *Mettl3*-CDS expression plasmid involved the cloning of the mouse *Mettl3* open reading frame of full-length (NM 019721) into the retroviral vector pMYs-IRES-GFP, which was generously provided by Dr. Xiaoming Feng from the Tianjin Hematology Hospital, Chinese Academy of Medical Sciences. To produce the catalytically inactive variant of *Mettl3*, the mutant *Mettl3*-CDS (D395A and W398A) [28] was amplified via PCR and subsequently cloned into the pMYs-IRES-GFP vector. The exact location of the mutant site is depicted in Supplementary Fig. S4D. Using PCR amplification, the complete-length cDNA of the mouse *Akt1* (NM 009652) was obtained and then cloned into the pMYs-IRES-GFP vector. For knockdown experiments, the sense and antisense sequences of short hairpin RNA (shRNA) against *Mettl3* (5′-GCTGCACTTCAGACGAATTAT-3′ and 5′-GCTCAATATACCAGTGCTACA-3′, respectively) or *Akt1* (5′-GCAGTGGACCACAGTCATTGA-3′ and 5′-GGAAGGTGATTCTGGTGAAAG-3′, respectively) and the construction of non-specific shRNA were designed and cloned into a lentiviral vector pLKO.1-EGFP-puro purchased from Fuzhou Sunya Biotechnology Co., Ltd, China. For retrovirus production, 2 μg of pMYs vectors were combined with 1 μg of pCL-Eco. Similarly, for lentivirus production, 2 μg of pLKO.1 vectors was combined with 1.5 μg of psPAX2 and 0.5 μg of pMD2.G. These plasmid mixtures were used for transient transfection of 293T cells which were cultured in 35-mm cell culture plate and had reached 80–90% confluence. Lipofectamine 3000 (Invitrogen) was utilized for the transfection process. The supernatant containing viral particles was collected in two separate batches within collection intervals spanning 48–72 h post transfection. Following this, the OP9 cells were subjected to retroviral or lentiviral transduction, and polybrene was added to the culture at a concentration of 10 μg/mL. The transduction process was allowed to proceed for 72 h. Inverted fluorescence microscopy was employed to observe the transduction efficiency, which was determined to be greater than 85%. The impact of gene overexpression and knockdown was verified using qPCR and western blot analysis.

### RNA-binding protein immunoprecipitation (RIP) assay

Magna RIP Kits (Cat# 17–700, Millipore Sigma Co., Ltd., Burlington, MA, USA) were employed to immunoprecipitate the RNA-binding proteins following the manufacturer’s directions. The harvested cells were lysed utilizing RIP lysis buffer. These cells were then incubated with anti-m^6^A (Cat# 202008, Synaptic Systems GmbH, Gottingen, Germany), the anti-METTL3 (Cat# 86132, Cell Signaling Technology, Danvers, MA, USA), and anti-IgG antibodies (Cat# PP64B, Millipore Sigma Co., Ltd.) at 4 °C overnight. TRIzol (Invitrogen) reagent was utilized to obtain RNA complexes, followed by qPCR.

### Western blot analysis

Following the manufacturer’s direction, proteins were isolated from cells using RIPA Lysis Buffer (Cat# P0013B, Beyotime, Nanjing, Jiangsu, China). The sodium dodecyl-sulfate polyacrylamide gel electrophoresis was employed to isolate proteins transferred onto polyvinylidene fluoride membranes (Cat# IPVH00005, Millipore Sigma Co., Ltd.). Following the blocking of the membranes with 5% milk, they were subjected to incubation with primary antibodies against METTL3 (Cat# 86132, Cell Signaling Technology), AKT1 (Cat# 75692, Cell Signaling Technology), p-AKT1 (Ser473) (Cat# 4060S, Cell Signaling Technology), AKT (Cat# 4685, Cell Signaling Technology), and β-actin (Cat# AF0003, Beyotime). The p-AKT1 (Ser473) antibody specifically recognizes AKT1 protein levels when phosphorylated at Ser473. The secondary antibodies used in this study were either HRP-conjugated goat anti-mouse (Cat# A0216, Beyotime) or rabbit IgG (H + L) (Cat# A0208, Beyotime), depending on the specific context of the experiments. The detection of chemiluminescence signals was performed employing the BeyoECL Star Kit (Beyotime) and ChemiDoc Touch system (Bio-Rad, Hercules, CA, USA) after the treatment of membranes with horseradish peroxidase-conjugated secondary antibodies.

### Flow cytometry (FCM) analysis

The AML cells was distinguished and quantified by directly identifying cells that were YFP-positive (YFP^+^), referring to them as the tumor cell population. For analysis of the proportion of YFP^+^ cells, single-cell suspensions were prepared from the spleen, liver, and BM. An appropriate amount of cell suspension was taken and centrifuged at 1500 rpm for 5 min. Cells were then washed twice with PBS containing 2% FBS, followed by lysis of red blood cells using a lysis buffer (Cat# R1010, Solarbio) for 10 min at 4 °C. PB samples were directly lysed in the same manner. Finally, the cells were washed twice in PBS containing 2% FBS. Flow cytometry data were acquired on a flow cytometer (BD Biosciences FACS Celesta, San Jose, CA, USA), and the software FlowJo 10.7.2 was used to analyze the results.

### Histomorphometric analyses and quantification of the number of AML cells in hematoxylin and eosin (H&E) staining

The primary tissues were treated with 4% paraformaldehyde (PFA), followed by embedding in paraffin (SAV LP) and sectioning into 5 μm slices for H&E staining. Following the manufacturer’s directions, H&E staining was performed utilizing an H&E kit (Cat# G1003, Servicebio, Wuhan, Hubei, China). ImageJ software was used to perform a quantitative analysis of AML cells in H&E-stained images of liver, spleen and BM. These cells were identified by their distinctive characteristics, including larger and irregular cell morphology, irregular nuclei, and reduced cytoplasm. Statistical analysis was conducted from each sample, drawing from observations taken in five different fields of view. The AML cell count was expressed as the number of AML cells per square millimeter (/mm^2^).

### Quantification of the number and area of adipocyte in bone marrow

To quantify the number and area of adipocytes in the distal marrow of femora from mice, a combination of AdipoCount and ImageJ software was employed as previously described [29]. For each sample, statistical analysis was performed based on observations from three different fields of view. The adipocyte number was expressed as the number of adipocytes per square millimeter (/mm^2^), and the percentage of adipocyte area was presented as a percentage (%) of adipocyte area per tissue area.

### Immunohistochemical staining

Before sectioning (5 μm), the dissected femurs were treated with 4% polyoxymethylene and subjected to decalcification in 10% ethylenediaminetetraacetic acid for 2 weeks. The antigen retrieval process was executed by immersing the sample in a sodium citrate solution at 99 °C for a duration of 20 min. Next, the sections were uniformly covered with 3% BSA at room temperature for 30 min and incubated with the primary antibody targeting fatty acid-binding protein 4 (FABP4; Cat# ab92501, Abcam, Cambridge, UK) at 4 °C overnight. FABP4 is a FABP family member and is highly expressed in adipocytes [30]. The next day, the sections were incubated with the corresponding secondary antibody (Cat# ab6721; Abcam) for 50 min at room temperature, followed by DAB color development processed with a DAB chromogenic reagent for the histochemical kit (Cat# G1212, Servicebio). The color developing time was controlled under the microscope. The positive color was brown and yellow, and the sections were rinsed with water to terminate the color development. Finally, the sections were stained with hematoxylin for ~3 min.

### Statistical analysis

All data were presented as means ± standard deviation (SD). Depending on normally distributed data, either the two-tailed unpaired Student’s *t* test or a one-way analysis of variance (ANOVA) with Dunnett’s multiple comparisons test was employed to assess differences between groups. The variance was similar between the groups that were being statistically compared. Survival analysis was performed using Kaplan–Meier survival curves for the mice, and statistical significance was determined using the log-rank (Mantel–Cox) test. All *P* values were calculated using GraphPad Prism 9.4.1, and statistical significance was defined as *P* < 0.05.

## Results

### Deletion of *Mettl3* in MSCs induces high marrow adiposity in mice

**Fig. 1:**
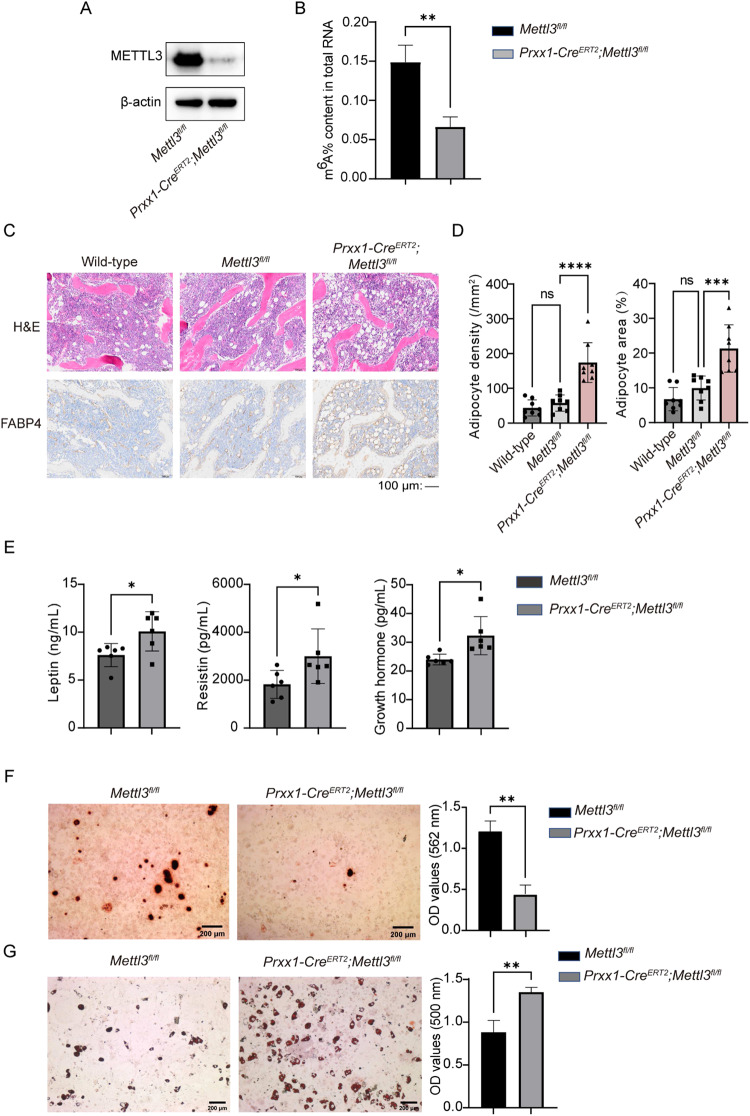
Deletion of *Mettl3* in MSCs induces high marrow adiposity in mice. **A** Western blot analysis of METTL3 in BMMSCs isolated from mice. **B** Detections of m^6^A levels in total RNA in BMMSCs from mice using EpiQuik m^6^A RNA Methylation Quantification Kits. **C** Representative images of H&E staining and FABP4 immunohistochemical staining of the distal femora from mice. Scale Bars: 100 μm. **D** Quantification of adipocyte number and area per tissue area in the distal marrow (*n* = 8). **E** ELISA examined the contents of supernatant adipokines in bone marrow rinses of mice (*n* = 6). **F** Representative images and quantitative analyses of Alizarin Red Solution (ARS) staining of BMMSCs isolated from mice. Scale Bars: 200 μm. **G** Representative images and quantitative analyses of ORO staining of BMMSCs isolated from mice. Scale Bars: 200 μm. The data are presented as mean ± SD. The two-tailed unpaired Student’s *t* test was used to compare two groups and one-way ANOVA with Dunnett’s multiple comparisons test was used for data with more than two groups. *P < 0.05, **P < 0.01, ***P < 0.001, ****P < 0.0001, ns not significant.

Conditional homozygous *Mettl3* knockout (*Prrx1-Cre*^*ERT2*^*;Mettl3*^*fl/fl*^) mice were produced by crossing *Mettl3*^*fl/fl*^ mice with *Prrx1-Cre*^*ERT2*^*;Mettl3*^*fl/+*^ mice (Supplementary Fig. S1A). A PCR analysis of genomic DNA from mouse tails was used to accurately identify these transgenic mice genotypes (Supplementary Fig. S1B). Western blot analysis was conducted to verify the effective deletion of *Mettl3* in BMMSCs (Fig. 1A), and the m^6^A levels were found to be reduced in BMMSCs from *Mettl3* conditional knockout mice with the mean m^6^A level being 0.07% compared to 0.15% in the control group (*P* = 0.0047) (Fig. 1B). H&E staining and FABP4 immunohistochemical analyses exhibited an elevated bone marrow adipose tissue (MAT) accumulation in the *Prrx1-Cre*^*ERT2*^*;Mettl3*^*fl/fl*^ mice (Fig. 1C). Compared with *Mettl3*^*fl/fl*^ mice, *Prrx1-Cre*^*ERT2*^*;Mettl3*^*fl/fl*^ mice showed a greater marrow adipocyte number (3.0-fold increase) and area (2.1-fold increase). However, there was no significant difference in the number (*P* = 0.6844) and area (*P* = 0.3272) of bone marrow adipocyte between wild-type mice and *Mettl3*^*fl/fl*^ mice (Fig. 1D). The levels of adipokines (including leptin, resistin and growth hormone) were found to be elevated in the supernatant of the quantitative bone marrow rinse solution from *Prrx1-Cre*^*ERT2*^*;Mettl3*^*fl/fl*^ mice. Specifically, the mean concentration was higher for leptin (10.08 versus 7.61 ng/mL, *P* = 0.0291), resistin (3003.00 versus 1825.00 pg/mL, *P* = 0.0476), and growth hormone (mean 32.20 versus 24.00 pg/mL, *P* = 0.0146), respectively **(**Fig. 1E). In addition, to assess the in vitro osteogenic and adipogenic potential of BMMSCs, they were isolated from *Mettl3*^*fl/fl*^ and *Prrx1-Cre*^*ERT2*^*;Mettl3*^*fl/fl*^ mice. The results showed that *Mettl3* deletion in BMMSCs caused a decrease in calcium mineralization, with a mean optical density (OD) value 0.44 compared to 1.20 in the control group (*P* = 0.0014). In addition, there was an increase in Oil Red O (ORO) staining intensity, indicated by a mean OD value of 1.35 in the *Mettl3*-deleted group versus 0.88 in the control group (*P* = 0.0052) (Fig. 1F, G). Overall, *Mettl3* conditional knockout in MSCs resulted in a change in the bone marrow microenvironment, characterized by high marrow adiposity in vivo.

### Deletion of *Mettl3* in MSCs accelerates AML progression in mice

**Fig. 2:**
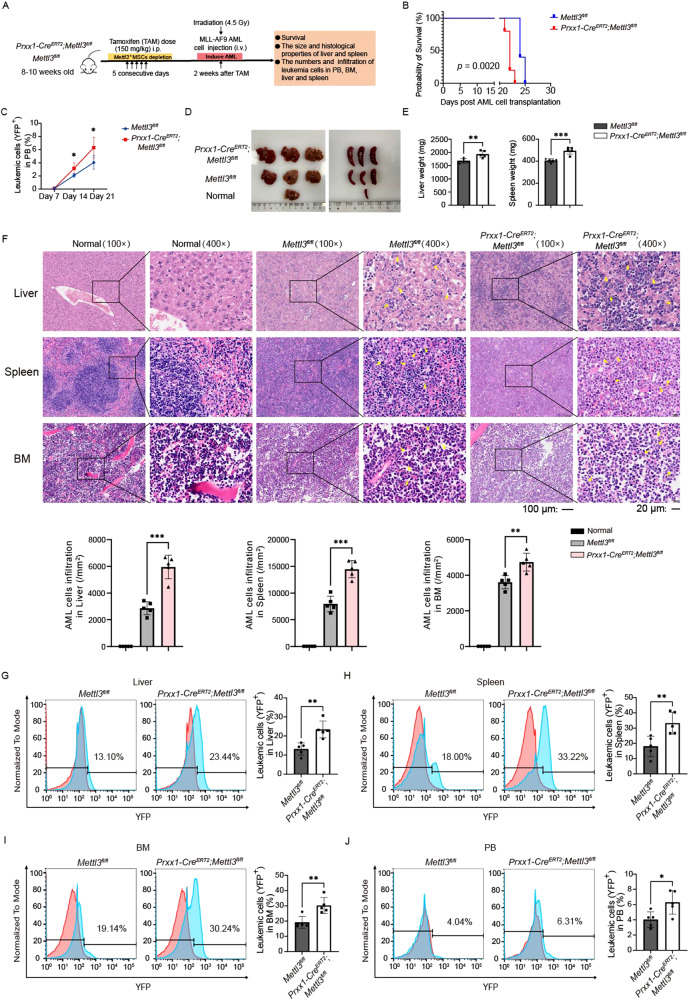
Deletion of *Mettl3* in MSCs before AML cell transplantation accelerates AML progression in mice. **A** Experimental design. 8–10 weeks-old *Mettl3*^*fl/fl*^ and *Prxx1-Cre*^*ERT2*^*;Mettl3*^*fl/fl*^ mice were given TAM injections for 5 consecutive days to induce the specific deletion of *Mettl3*^+^ MSCs. Two weeks later, both *Mettl3*^*fl/fl*^ and *Prxx1-Cre*^*ERT2*^*;Mettl3*^*fl/fl*^ mice that had been irradiated with 4.5 Gy were transplanted the MLL-AF9 AML cells to induce AML. **B** Survival curve of mice after transplantation (*n* = 5). **C** The percentage of YFP^+^ cells in PB was measured weekly by orbital blood after transplantation (*n* = 5). **D** Representative pictures of livers and spleens obtained from normal mice, *Mettl3*^*fl/fl*^ and *Prxx1-Cre*^*ERT2*^*;Mettl3*^*fl/fl*^ AML mice. **E** Comparison of the weights of livers and spleens of the *Mettl3*^*fl/fl*^ and *Prxx1-Cre*^*ERT2*^*;Mettl3*^*fl/fl*^ AML mice (*n* = 5). **F** Representative images of H&E staining (top) and the corresponding quantification of AML cells per square millimeter (bottom) for liver, spleen and BM sections from normal mice, *Mettl3*^*fl/fl*^ and *Prxx1-Cre*^*ERT2*^*;Mettl3*^*fl/fl*^ AML mice (*n* = 5). The yellow arrows point to AML cells. Scale bars: 100 μm, ×100 magnification. Scale Bars: 20 μm, ×400 magnification. **G**–**J** Representative FCM profiles (red, normal control; blue, *Mettl3*^*fl/fl*^ or *Prxx1-Cre*^*ERT2*^*;Mettl3*^*fl/fl*^ AML mice) and quantification of the percentage of YFP^+^ AML cells (right) in liver (**G**), spleen (**H**), BM (**I**), and PB (**J**) from *Mettl3*^*fl/fl*^ and *Prxx1-Cre*^*ERT2*^*;Mettl3*^*fl/fl*^ mice after transplantation (*n* = 5). The data are presented as mean ± SD. Differences were determined using the two-tailed unpaired Student’s *t* test. *P < 0.05, **P < 0.01, ***P < 0.001.

Altered *Mettl3* expression in MSCs affects BM niche formation [24], suggesting that the gene has a potential function in the symptomatic AML start and development. To explore this, we used an MLL-AF9 AML model induced by transplantation in transgenic irradiated mice. Two weeks after *Mettl3* deletion, we intravenously implanted YFP^+^ MLL-AF9 AML cells into irradiated (4.5 Gy) recipients (Fig. 2A). Notably, *Prrx1-Cre*^*ERT2*^*;Mettl3*^*fl/fl*^ mice had a significantly decreased survival time and shorter symptomatic AML latency than control mice (median survival 22 versus 24 days, *P* = 0.0020) (Fig. 2B). In *Mettl3*^*fl/fl*^ mice and *Prrx1-Cre*^*ERT2*^*;Mettl3*^*fl/fl*^ mice, the symptomatic AML development was detected at 24–25 days and 21–23 days after transplantation, respectively. FCM analysis revealed a more rapid increase in the total engraftment of MLL-AF9 AML cells in the PB of *Prrx1-Cre*^*ERT2*^*;Mettl3*^*fl/fl*^ mice compared to *Mettl3*^*fl/fl*^ mice after transplantation (mean increase of 3.12% versus 2.09% from day 7 to day 14, and mean increase of 3.14% versus 1.95% from day 14 to day 21, respectively) (Fig. 2C). Disease progression was accelerated following *Mettl3* deletion, as evidenced by the faster development of hepatosplenomegaly (Fig. 2D, E), increased AML cell infiltration in the liver (mean 5956 AML cells/mm^2^ versus 2864 AML cells/mm^2^ in H&E staining, *P* = 0.0001; 23.44 ± 4.46 versus 13.10 ± 3.23% of FCM analysis, *P* = 0.0030), spleen (mean 14,472 AML cells/mm^2^ versus 7960 AML cells/mm^2^ in H&E staining, *P* = 0.0002; 33.22 ± 7.21% versus 18.00 ± 6.73% of FCM analysis, *P* = 0.0087) (Fig. 2F–H), BM (mean 4739 AML cells/mm^2^ versus 3608 AML cells/mm^2^ in H&E staining, *P* = 0.0036; 30.24 ± 5.22% versus 19.14 ± 3.94% of FCM analysis, *P* = 0.0053) (Fig. 2F, I) and higher circulating leukemic blasts in the PB (6.31 ± 1.55% versus 4.04 ± 1.03%, *P* = 0.0259) (Fig. 2J) of *Prrx1-Cre*^*ERT2*^*;Mettl3*^*fl/fl*^ mice in contrast to those of control mice. In addition, we observed that *Mettl3* deletion in MSCs after AML cells transplantation accelerated AML progression in mice.

Mice were treated with TAM to precisely deplete *Mettl3*^+^ cells one day after YFP^+^ MLL-AF9 AML cell transplantation (Supplementary Fig. S2A). Depletion of *Mettl3*^+^ cells upon the development of AML significantly shortened mouse survival (median survival 17 versus 21 days, *P* = 0.0018) (Supplementary Fig. S2B). The weekly percentage increase in AML cells in *Prrx1-Cre*^*ERT2*^*;Mettl3*^*fl/fl*^ mice were greater than that in control mice (mean increase of 3.83% versus 1.25% from day 7 to day 14, and mean increase of 8.46% versus 1.32% from day 14 to day 17, respectively) (Supplementary Fig. S2C). *Prrx1-Cre*^*ERT2*^*;Mettl3*^*fl/fl*^ mice developed AML faster, and hepatosplenomegaly was more severe (Supplementary Fig. S2D, E), with greater AML cell infiltration into the liver (mean 4148 AML cells/mm^2^ versus 2080 AML cells/mm^2^ in H&E staining, *P* = 0.0005; 10.65 ± 2.35% versus 5.79 ± 0.63% of FCM analysis, *P* = 0.0021), spleen (mean 12,400 AML cells/mm^2^ versus 7120 AML cells/mm^2^ in H&E staining, *P* = 0.0012; 23.82 ± 3.66% versus 14.08 ± 4.55% of FCM analysis, *P* = 0.0058) (Supplementary Fig. S2F–H) and BM (mean 5660 AML cells/mm^2^ versus 3484 AML cells/mm^2^ in H&E staining, *P* = 0.0091; 34.28 ± 5.97% versus 13.54 ± 7.06% of FCM analysis, *P* = 0.0010) (Supplementary Fig. S2F, I). In addition, circulating leukemic blasts in the PB (12.29 ± 2.80% versus 2.57 ± 0.67%, *P* < 0.0001) (Supplementary Fig. S2J) were more pronounced in *Prrx1-Cre*^*ERT2*^*;Mettl3*^*fl/fl*^ mice. These findings imply that *Mettl3* deletion in MSCs accelerates AML progression, regardless of whether it is induced before or after AML cell transplantation, and that METTL3 in MSCs may serve as a repressor of AML development.

### Deletion of *Mettl3* in MSCs decreases the AML cells’ sensitivity to chemotherapy in vivo

**Fig. 3:**
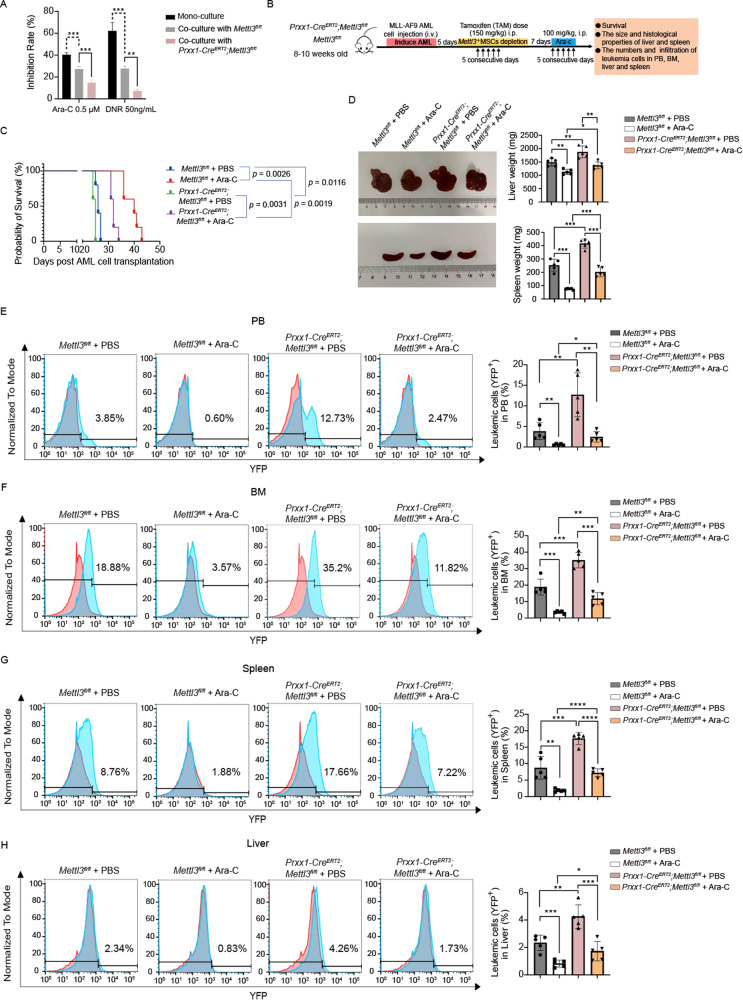
Deletion of *Mettl3* in MSCs decreases the AML cells’ sensitivity to chemotherapy in vivo. **A** Primary MLL-AF9 AML cells were co-cultured with the adipo-induced BMMSCs isolated from *Mettl3*^*fl/fl*^ and *Prxx1-Cre*^*ERT2*^*;Mettl3*^*fl/fl*^ mice, and the inhibition rate was analyzed using the CCK-8. **B** Experimental design. Nonirradiated recipients were transplanted with 2 × 10^5^ MLL-AF9 AML cells. After 5 days, *Mettl3* depletion was induced by TAM via intraperitoneal injection. *Mettl3*^*fl/fl*^ or *Prxx1-Cre*^*ERT2*^*;Mettl3*^*fl/fl*^ AML mice were randomly assigned to two treatment groups, one receiving chemotherapy Ara-C and the other receiving vehicle (PBS) injections for 5 days, beginning one week after TAM induction. **C** Kaplan–Meier survival curve of AML mice in the following groups: *Mettl3*^*fl/fl*^ + PBS (blue, *n* = 5), *Mettl3*^*fl/fl*^ + Ara-c (red, *n* = 5), *Prxx1-Cre*^*ERT2*^*;Mettl3*^*fl/fl*^ + PBS (green, *n* = 5), and *Prxx1-Cre*^*ERT2*^*;Mettl3*^*fl/fl*^ + Ara-c (purple, *n* = 5). **D** Comparison of the sizes and weights of livers and spleens among the four groups (*n* = 5). **E**–**H** Representative FACS profiles (red, normal control; blue, *Mettl3*^*fl/fl*^ or *Prxx1-Cre*^*ERT2*^*;Mettl3*^*fl/fl*^ AML mice) and quantification of the percentage (right) of YFP^+^ AML cells in PB (**E**), BM (**F**), spleen (**G**) and liver (**H**) after transplantation (*n* = 5). The data are presented as mean ± SD. Differences were determined using the two-tailed unpaired Student’s *t* test for data with two groups and one-way ANOVA with Dunnett’s multiple comparisons test was used for data with more than two groups. *P < 0.05, **P < 0.01, ***P < 0.001, ****P < 0.0001.

Next, we aimed to discover the *Mettl3* deficiency impact in BMMSCs in the response to chemotherapy, we isolated BMMSCs from *Mettl3*^*fl/fl*^ mice and *Prrx1-Cre*^*ERT2*^*;Mettl3*^*fl/fl*^ mice 2 weeks after *Mettl3* deletion. The cells were stimulated to undergo adipogenesis and subsequently subjected to co-culture with primary MLL-AF9 AML cells. A CCK-8 assay revealed that primary MLL-AF9 AML cells co-cultured with *Mettl3* knockout BMMSCs exhibited reduced sensitivity to the chemotherapeutic drugs Ara-C and DNR (Fig. 3A). We then assessed whether the in vitro chemotherapeutic effects of *Mettl3* deletion in BMMSCs would translate into a tumor response in vivo by intravenously injecting 2 × 10^5^ MLL-AF9 AML cells into nonirradiated *Prrx1-Cre*^*ERT2*^*;Mettl3*^*fl/fl*^ mice and *Mettl3*^*fl/fl*^ mice to establish a systemic AML model. We randomly assigned the mice to four groups and administered a daily intraperitoneal injection of either vehicle control or Ara-C (100 mg/kg/day) for 5 consecutive days (Fig. 3B). We observed that *Mettl3* deletion in BMMSCs conferred chemoresistance to *Prrx1-Cre*^*ERT2*^*;Mettl3*^*fl/fl*^ mice as evidenced by their shorter survival times (median survival of 32 days for *Prrx1-Cre*^*ERT2*^*;Mettl3*^*fl/fl*^ + Ara-C group and 40 days for *Mettl3*^*fl/fl*^ + Ara-C group, respectively) (Fig. 3C). Furthermore, the livers and spleens of *Prrx1-Cre*^*ERT2*^*;Mettl3*^*fl/fl*^ mice remained larger than those of *Mettl3*^*fl/fl*^ mice 5 days post-Ara-C treatment (Fig. 3D). Likewise, total AML cell engraftment was significantly higher in the PB (2.47 ± 1.28% versus 0.60 ± 0.16%, *P* = 0.0121) (Fig. 3E), BM (11.82 ± 3.70% versus 3.57 ± 0.75%, *P* = 0.0012) (Fig. 3F), spleen (7.22 ± 1.16% versus 1.88 ± 0.45%, *P* < 0.0001) (Fig. 3G) and liver (1.73 ± 0.70% versus 0.83 ± 0.26%, *P* = 0.0265) (Fig. 3H) of the *Mettl3*-depleted mice than in nondepleted mice after Ara-C treatment. These data suggest that *Mettl3* deletion in BMMSCs enhances their supportive and protective effects in AML.

### METTL3 deletion reduces AML chemosensitivity by inducing adipogenic differentiation of OP9 cells

**Fig. 4:**
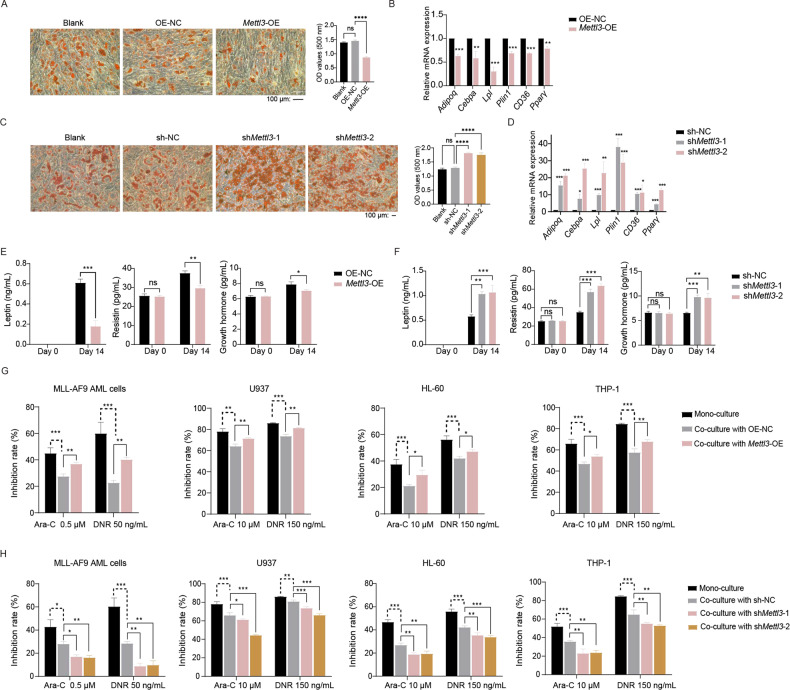
METTL3 deletion reduces AML chemosensitivity by inducing adipogenic differentiation of OP9 cells. **A** Representative images and quantification of ORO staining in *Mettl3* overexpression cells after 14 days of adipogenic induction. Scale bar, 100 µm. **B** qPCR analysis of the expression of adipogenic marker genes, *Adipoq*, *Cebpa*, *Lpl*, *Plin1*, *CD36*, and *Pparγ* in *Mettl3* overexpression cells under adipogenic conditions. **C** ORO staining of *Mettl3* knockdown cells following adipogenic induction. Scale bar, 100 µm. Absorbance at OD_500_ was determined for ORO staining in isopropanol at room temperature. **D** qPCR analysis of adipogenic lineage–associated gene expression after induction of differentiation in *Mettl3* knockdown cells. **E** ELISA detection of adipokine concentration in the culture medium before induction of differentiation and on day 14 of *Mettl3* overexpression cells. **F** ELISA detection of adipokine concentration in the culture medium before induction of differentiation and on day 14 of *Mettl3* knockdown cells. **G** After adipogenic differentiation of *Mettl3* overexpression cells, AML cells were co-cultured with them for 24 h to test their chemoresistance to Ara-C and DNR. **H** After adipogenic differentiation of *Mettl3* knockdown cells, AML cells were co-cultured with them for 24 h to test their chemoresistance to Ara-C and DNR. Blank, blank control; OE-NC, negative control of overexpression; *Mettl3-*OE, overexpression of mouse *Mettl3*; sh-NC, negative control of knockdown; sh*Mettl3*-1 and sh*Mettl3*-2, independent shRNAs targeting mouse *Mettl3*. The data are presented as mean ± SD. The two-tailed unpaired Student’s *t* test was used to compare two groups and one-way ANOVA with Dunnett’s multiple comparisons test was performed for multiple groups. *P < 0.05, **P < 0.01, ***P < 0.001, ****P < 0.0001, ns not significant.

To further examine the METTL3 function in the MSCs adipogenic differentiation, we used OP9 cells, which are commonly used as a model for MSCs [31]. Gene editing was used to modulate METTL3 expression in OP9 cells, and the effectiveness of overexpression and knockdown was approved by western blot, qPCR, and m^6^A RNA analyses (Supplementary Fig. S3). The overexpression of *Mettl3* caused a significant reduction in OP9 cell adipogenesis and the downregulation of adipogenic factors, such as *Adipoq*, *Cebpa*, *Lpl*, *Plin1*, *CD36*, and *Pparγ* (Fig. 4A, B), while the knockdown of *Mettl3* promoted OP9 cell adipogenesis and increased the expression of adipogenic factors (Fig. 4C, D). We also measured the levels of secreted adipokines (leptin, resistin, and growth hormone) before and after the induction of differentiation in overexpression and knockdown of *Mettl3* cells (Fig. 4E, F). The knockdown of *Mettl3* raised these adipokines levels after induction of differentiation for 14 days. We then co-cultured AML cells with differentiated adipocytes to determine whether METTL3 affects the AML cells’ sensitivity to chemotherapy through adipogenic differentiation. The overexpression of *Mettl3* improved the co-cultured AML cells’ sensitivity to chemotherapy, whereas the knockdown of *Mettl3* resulted in chemoresistance compared to sensitivity in the controls (Fig. 4G, H). These outcomes indicate that the depletion of *Mettl3* expression promotes adipogenesis of OP9 cells, thereby decreasing the AML cells’ sensitivity to chemotherapy.

### METTL3 promotes the *Akt1* expression in an m^6^A-dependent manner

**Fig. 5:**
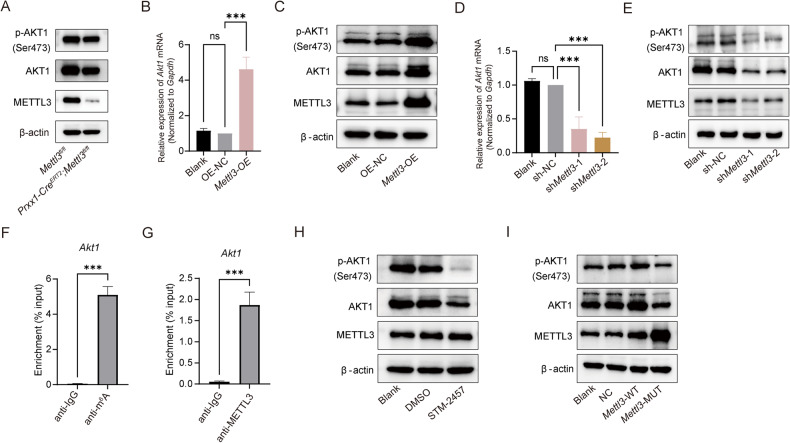
METTL3 promotes the expression of *Akt1* in an m^6^A-dependent manner. **A** Western blot analysis of METTL3, AKT1, and p-AKT1 (Ser473) in BMMSCs isolated from *Mettl3*^*fl/fl*^ and *Prxx1-Cre;Mettl3*^*fl/fl*^ mice. **B** qPCR analysis of *Akt1* mRNA levels after *Mettl3* overexpression. **C** Western blot analysis of METTL3, AKT1, and p-AKT1 (Ser473) protein levels after *Mettl3* overexpression. **D** qPCR analysis of *Akt1* mRNA levels after *Mettl3* knockdown. **E** Western blot analysis of METTL3, AKT1, and p-AKT1 (Ser473) protein levels after *Mettl3* knockdown. **F** MeRIP-qPCR analysis using anti-IgG and anti-m^6^A antibodies to identify *Akt1* mRNA modified by m^6^A. **G** qPCR for *Akt1* mRNA was performed on RNA-IP with the anti-IgG and anti-METTL3 antibodies. **H** Western blot analysis of METTL3, AKT1, and p-AKT1 (Ser473) protein levels in OP9 cells treated with STM2457 for 24 h. **I** Western blot analysis of METTL3, AKT1, and p-AKT1 (Ser473) in OP9 cells transfected with NC, *Mettl3*-WT and *Mettl3*-MUT plasmid. NC, negative control; *Mettl3*-WT, *Mettl3* wild type; *Mettl3*-MUT, *Mettl3* mutation. The data are presented as mean ± SD.The two-tailed unpaired Student’s *t* test was used to compare two groups and one-way ANOVA with Dunnett’s multiple comparisons test was performed for multiple groups. ***P < 0.001, ns not significant.

Next, we performed western blot analysis to detect the molecular pathway underlying the inhibitory impact of METTL3 on MSCs adipogenic differentiation. We discovered that the AKT1 and p-AKT1 (Ser473) expression levels were lower in BMMSCs isolated from *Prrx1-Cre*^*ERT2*^*;Mettl3*^*fl/fl*^ mice than in control cells (Fig. 5A). Furthermore, *Mettl3* overexpression in OP9 cells increased *Akt1* mRNA levels and AKT1 and p-AKT1 (Ser473) protein levels (Fig. 5B, C). In contrast, *Mettl3* knockdown decreased *Akt1* mRNA levels and AKT1 and p-AKT1 (Ser473) protein levels (Fig. 5D, E). Nevertheless, we did not detect any variation in the relative p-AKT1 (Ser473) and AKT1 expression levels between cells with overexpression or knockdown of *Mettl3* and the control group (Supplementary Fig. S4A, B). Since METTL3 is an RNA methyltransferase that dynamically controls m^6^A modification, we hypothesized it influences *Akt1* expression via m^6^A methylation. To evaluate this hypothesis, we conducted RNA immunoprecipitation combined with qPCR (RIP-qPCR) and found that *Akt1* mRNA was modified by m^6^A and bound to METTL3 (Fig. 5F, G). To determine whether METTL3 mediates AKT1 protein expression in an m^6^A-dependent manner, we treated cells with STM2457, an inhibitor of METTL3 catalytic activity, or the MUT-*Mettl3* plasmid, a catalytically inactive form of METTL3. After applying different concentrations of STM2457 to OP9 cells, an m^6^A-level analysis revealed that 15 μM was the most suitable concentration (Supplementary Fig. S4). As expected, the inhibitor and *Mettl3*-MUT significantly decreased AKT1 and p-AKT1 (Ser473) protein levels (Fig. 5H, I), indicating that METTL3 modulated the AKT1 expression in a methylation-dependent manner.

### METTL3 regulates OP9 cells adipogenic differentiation via the AKT1 signaling pathway

**Fig. 6:**
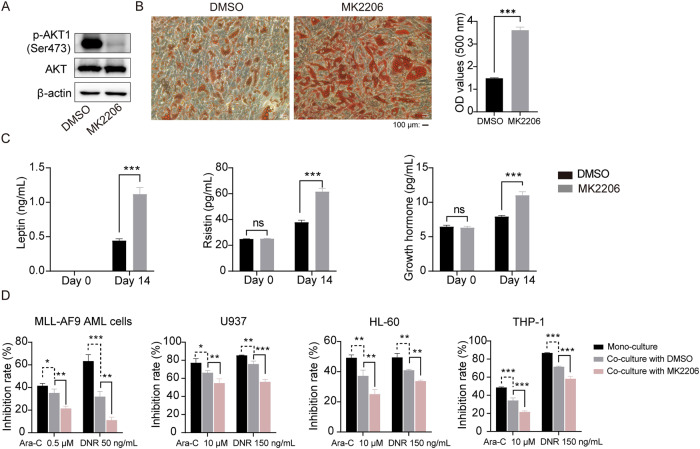
AKT inhibitor MK2206 mediates the adipogenesis level of OP9 cells. **A** Western blot analysis of AKT and p-AKT1 (Ser473) in OP9 cells treated with MK2206, an AKT inhibitor. **B** ORO staining of DMSO and MK2206 treated cells after adipo-induced for 14 days. Scale bar, 100 μm. **C** ELISA detected the concentration of adipokines in the culture medium before induction of differentiation and on day 14 under treatment with MK2206. **D** After being treated with MK2206 for 18 h and adipo-induced for 14 days, co-culturing with AML cells for 24 h was performed to test the chemoresistance of AML cells to Ara-C and DNR. The data are presented as mean ± SD. The two-tailed unpaired Student’s *t* test was used to compare two groups and one-way ANOVA with Dunnett’s multiple comparisons test was performed for multiple groups. *P < 0.05, **P < 0.01, ***P < 0.001, ns not significant.

**Fig. 7:**
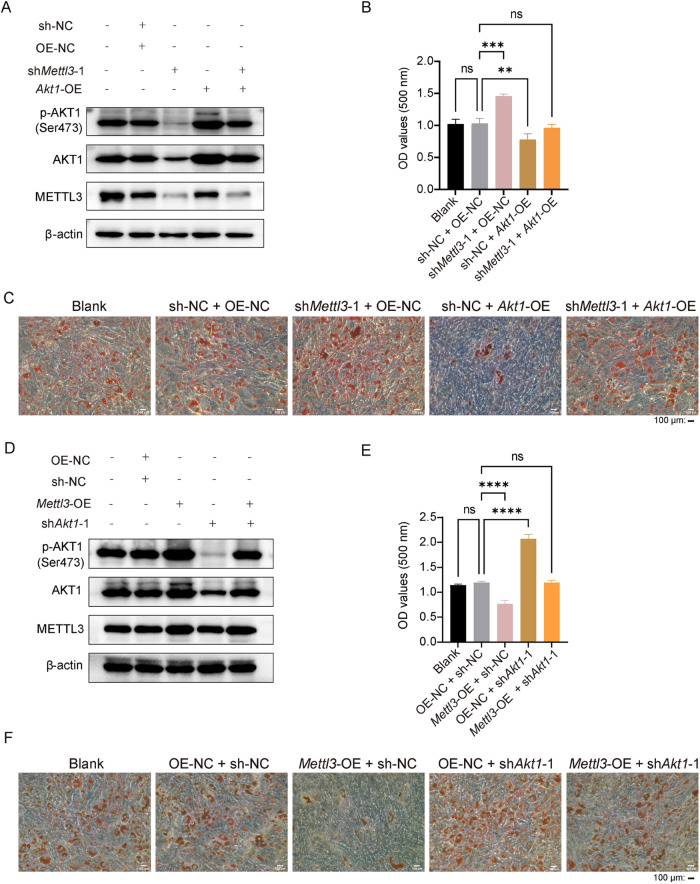
METTL3 regulates adipogenic differentiation of OP9 cells via the AKT1 signaling pathway. **A** *Akt1* overexpression rescued the *Mettl3* knockdown-induced decreases in AKT1 and p-AKT1 (Ser473) protein levels as measured by western blot analysis. **B** Absorbance at OD_500_ was determined in isopropanol at room temperature. **C** ORO staining of OP9 cells after adipo-induced for 14 days. Scale bar, 100 μm. **D** *Akt1* knockdown rescued the *Mettl3* overexpression-induced increased AKT1 and p-AKT1 (Ser473) protein levels as measured by western blot analysis. **E** Absorbance at OD_500_ was determined in isopropanol at room temperature. **F** ORO staining of OP9 cells after adipo-induced for 14 days. Scale bar, 100 μm. Blank, blank control; OE-NC, negative control of overexpression; *Mettl3-*OE, overexpression of mouse *Mettl3*; sh-NC, negative control of knockdown; sh*Mettl3*-1 and sh*Mettl3*-2 independent shRNAs targeting mouse *Mettl3*. The data are presented as mean ± SD. The one-way ANOVA with Dunnett’s multiple comparisons test was performed for multiple groups. **P < 0.01, ***P < 0.001, ****P < 0.0001, ns not significant.

We further determined whether AKT1 was necessary for the ability of METTL3 to suppress adipogenic differentiation in OP9 cells. We used MK2206, a highly potent and selective allosteric AKT inhibitor, to suppress p-AKT1 (Ser473) expression (Fig. 6A) and found that it significantly increased the adipogenic differentiation of OP9 cells (Fig. 6B). Notably, the MK2206 treatment also enhanced the secretion of adipokines (leptin, resistin and growth hormone) and inhibited the chemosensitivity of co-cultured AML cells (Fig. 6C, D). These outcomes led us to speculate that AKT1 participates in the regulation of adipogenesis via METTL3. To verify the AKT1 function in adipogenic differentiation, we infected cells with AKT1-expressing or sh*Akt1* lentiviral particles. We successfully achieved *Akt1* overexpression and *Akt1* knockdown at mRNA and protein levels, as verified by qPCR (Supplementary Fig. S5A, C) and western blot analysis (Supplementary Fig. S5B, D). We observed that *Akt1* overexpression reversed the *Mettl3* knockdown-induced decreases in AKT1 and p-AKT1 (Ser473) protein levels (Fig. 7A). ORO staining showed that *Mettl3* knockdown reversed the inhibition of adipogenesis induced by *Akt1* overexpression (Fig. 7B, C). Our data also confirmed that *Akt1* knockdown reversed the *Mettl3* overexpression-induced increases in AKT1 and p-AKT1 (Ser473) protein levels (Fig. 7D), and *Mettl3* overexpression reversed the up-regulation of adipogenesis induced by *Akt1* knockdown (Fig. 7E, F). These outcomes suggest that AKT1 is a METTL3 downstream target that affects the adipogenic differentiation and confers chemoresistance to AML cells.

## Discussion

Despite the known importance of BMMSCs in AML progression and resistance to chemotherapy, the precise mechanism by which BMMSCs participate in this process remains to be determined. This study showed that deleting *Mettl3* in mouse BMMSCs increased marrow adiposity and enhanced adipogenic potential, accelerating AML progression and conferring chemotherapy resistance.

Previous studies have suggested that AML BMMSCs have increased adipogenic potential [25, 32, 33] and that BMMSCs with increased adipogenesis elevated AML resistance to chemotherapy in vitro [25, 33]. However, the in vivo effects of this increased BMMSCs adipogenesis on AML progression and chemoresistance remain unknown. This is the first study to define the specific role of BMMSCs adipogenesis in the pathogenesis and treatment of AML in vivo.

Maintaining a precise balance between the osteogenic and adipogenic differentiation of BMMSCs is a highly regulated process that occurs in a spatiotemporal pattern [34]. The METTL3-mediated m^6^A modification of eukaryotic RNA is important in MSC pluripotent differentiation and development. The BMMSC-specific deletion of *Mettl3* in mice causes osteoporosis, increased marrow adiposity, and reduced osteogenic differentiation [24]. Another study showed that *Mettl3* knockdown in rat BMMSCs reduces alkaline phosphatase activity and mineralized nodule formation [35]. However, contradictory results have been obtained; for example, a study has shown that METTL3 activates MYD88 and NF-κB signaling, inhibiting osteogenic progression [36]. The various biological effects of METTL3 may be caused by its distinct downstream signals in different cell types and disease conditions.

The process of m^6^A methylation, catalyzed by METTL3, introduces a novel aspect to the modulation of gene expression at the posttranscriptional level. This mechanism can regulate various stages of the RNA life cycle, such as mRNA splicing, nuclear export, stability, and translation [37]. Previously, Liu et al. suggested that METTL3 mediates the *NR4A1* mRNA degradation via YTHDF2, thereby alleviating the NR4A1-induced transcriptional repression of AKT1 in cervical cancer [38]. METTL3-upregulated m^6^A methylation enhances the expression of AKT1 via *Akt1* mRNA stability that is mediated by YTHDF1 in arsenic carcinogenicity [39], whereas METTL3 induces m^6^A alteration in *Akt1* mRNA, which reduces AKT protein expression in human BMMSCs [25]. In contrast, our findings show that METTL3 upregulates expression of AKT1 in a way based on methylation in mouse BMMSCs, though the pathways underlying the differential modulation of AKT expression in human and mouse BMMSCs require further investigation. METTL3 deletion promotes adipogenesis in human and murine BMMSCs, leading to chemotherapy resistance.

Many studies have indicated that BMMSCs confer chemoresistance to leukemia cells by differentiating them into adipocytes. In both B-cell acute lymphoblastic leukemia and AML, obese patients with leukemia have poorer treatment responsiveness than that of non-obese patients [40]. Two frequently used chemotherapeutic drugs, DNR and vincristine, are aggressively sequestered and metabolized by adipocytes [41], and L-asparaginase, a major component of ALL therapy, is inhibited by glutamine secretion by adipocytes [42]. In addition, AML cells utilize fatty acid oxidation as a major energy source, and patients who achieve remission have a lower adipocyte content in their bone marrow than that of non-remission patients [43]. AML cells stimulate the generation of free fatty acids from bone marrow adipocytes by upregulating FABP4, which promotes AML blast survival [44]. Adipocytes also exert an anti-apoptotic effect on AML cells by raising fatty acid oxidation and upregulating the PPAR, CD36, and BCL2 expression [45]. Adipocytes secrete various factors, including leptin, platelet-derived growth factor, insulin-like growth factor 1, matrix metalloproteinase 11, interleukin 6, and stromal cell-derived factor 1 (SDF-1), and these are related to the AML cells migration, growth, and metastasis [46–48]. An important finding of our study is that *Mettl3*-deficient BMMSCs displayed increased leptin secretion compared to wild-type BMMSCs. Therefore, leptin produced by *Mettl3*-deficient BMMSCs may promote AML progression and chemoresistance; however, other factors mediate the effects of BMMSCs and require further analysis.

The intrinsic METTL3 function in AML has been described previously. Vu et al. reported that METTL3 knockout in AML cells promotes apoptosis and differentiation of AML cells, leading to delayed development of leukemia [21]. Yankova et al. discovered a METTL3 catalytic inhibitor that exhibits high efficacy and selectivity, significantly improving AML both in vitro and in vivo [49]. In addition, an investigation indicated that METTL3 expression in AML cells influenced AML chemoresistance and that its expression in individuals with AML was connected with poor treatment outcomes [22]. In the same year, Nagy et al. also discovered that METTL3 is overexpressed in AML patients and is associated with poor prognostic outcomes, including a failure to achieve hematological remission within 6 months after induction therapy [50]. These findings have promoted interest in the potential treatment effects of targeting METTL3 in AML cells. Our study is the first to shed light on the in vivo effect of METTL3 in BMMSCs on AML progression and chemosensitivity, though it is vital to note that METTL3 appears to affect AML progression differently in AML cells and BMMSCs. Therefore, tissue-specific targeting of METTL3 may be critical for achieving maximal efficacy.

In summary, our investigation offers new evidence of the in vivo function of METTL3 expressed by BMMSCs in suppressing AML progression and chemoresistance. METTL3 prevents the development of adipocyte-biased marrow microenvironments by promoting AKT1 expression. Thus, modulating METTL3 expression in BMMSCs may be a promising approach for suppressing AML progression and increasing sensitivity to chemotherapy.

### Supplementary information

Supplementary Figure S1

Supplementary Figure S2

Supplementary Figure S3

Supplementary Figure S4

Supplementary Figure S5

Supplementary Materials

Supplementary Table S1

Supplementary Table S2

Original Data File

checklist

## Data Availability

This study incorporates all data produced or analyzed throughout the research and is comprehensively presented in the published article and its supplementary information documents. Additional queries may be directed towards the corresponding author.
