# Supplementary figures and images for: Deletion of Mettl3 in mesenchymal stem cells promotes acute myeloid leukemia resistance to chemotherapy
Source: Cell Death Dis. 2023 Dec 5;14(12):796. doi: 10.1038/s41419-023-06325-7 (PMC10698052; doi:10.1038/s41419-023-06325-7)

A

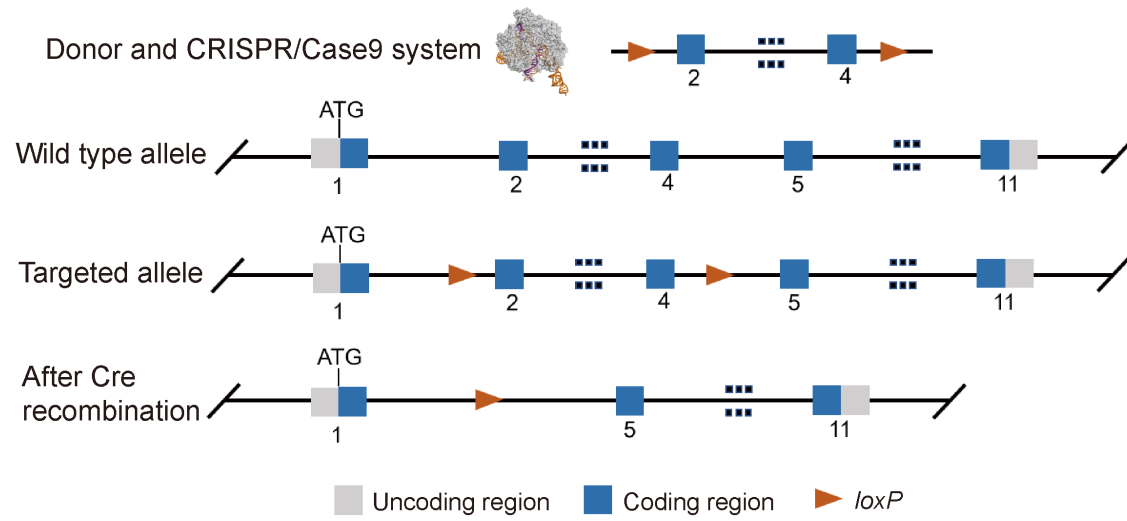

B

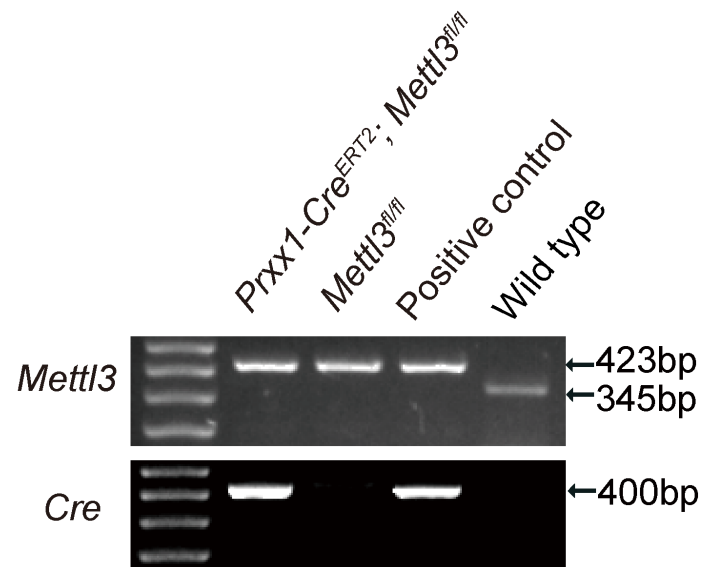

Supplementary Figure S1

Supplement: Supplementary file 1 — Supplementary Figure S1 [file 41419_2023_6325_MOESM1_ESM.pdf]

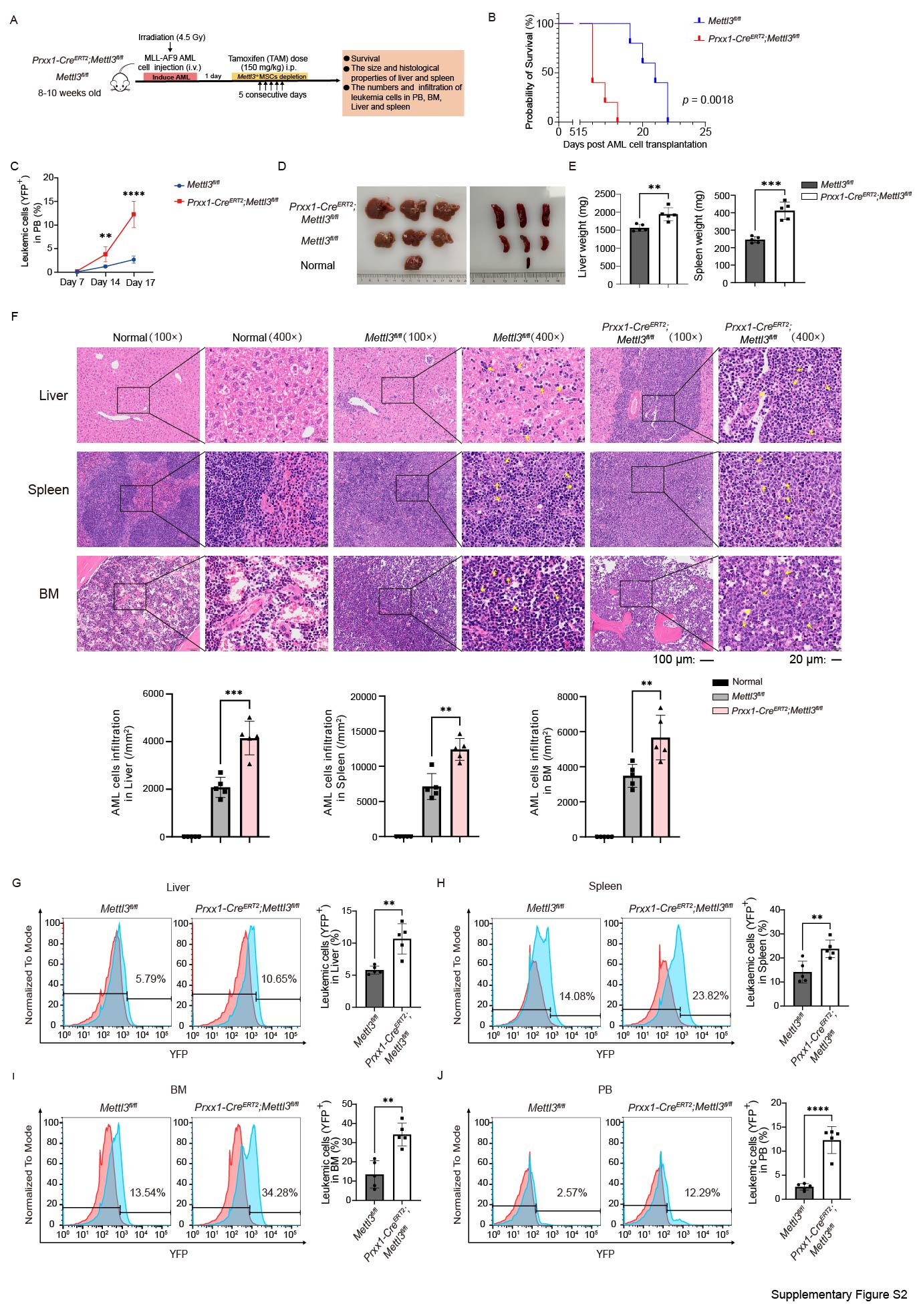

Supplement: Supplementary file 2 — Supplementary Figure S2 [file 41419_2023_6325_MOESM2_ESM.jpg]

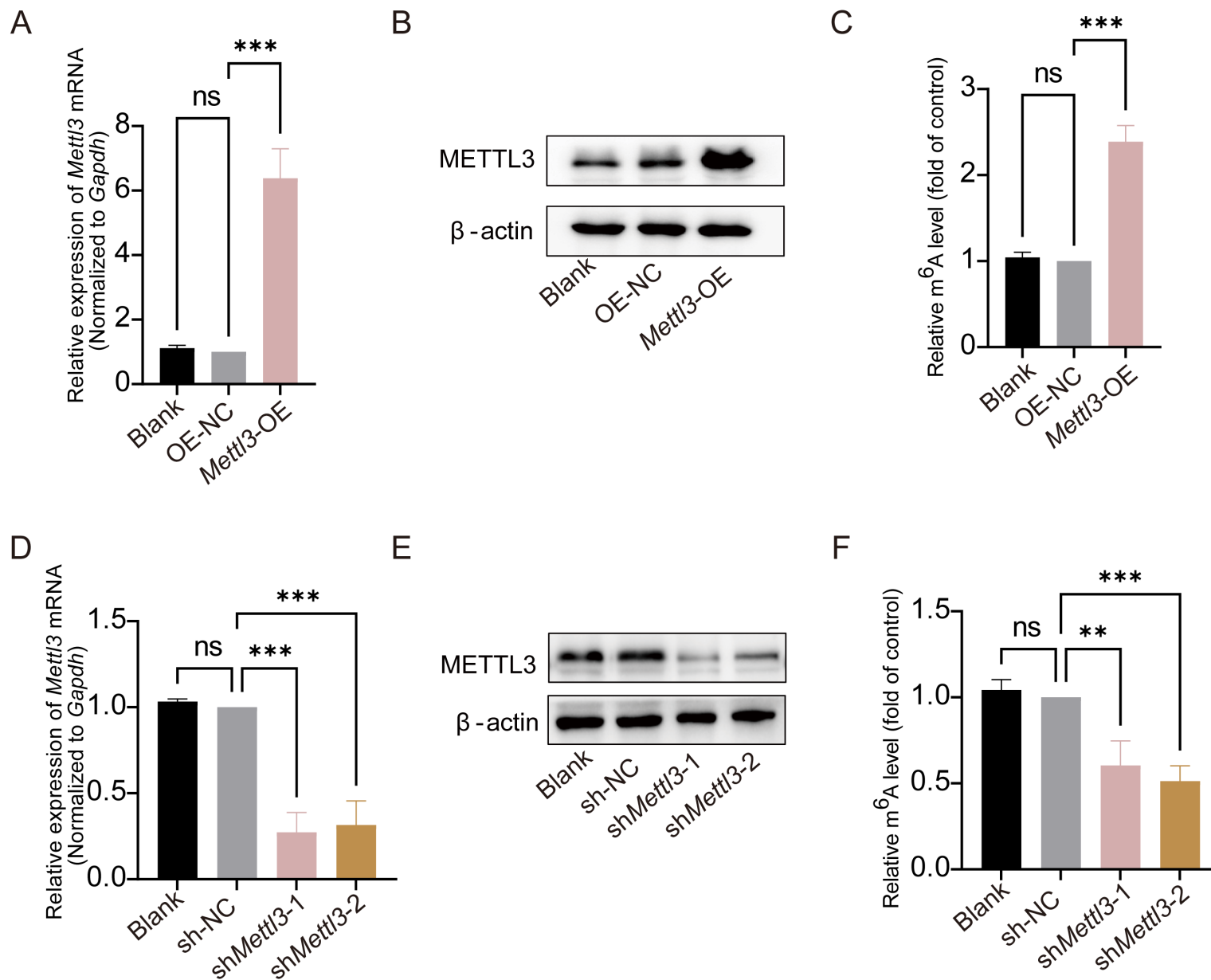

Supplementary Figure S3

Supplement: Supplementary file 3 — Supplementary Figure S3 [file 41419_2023_6325_MOESM3_ESM.pdf]

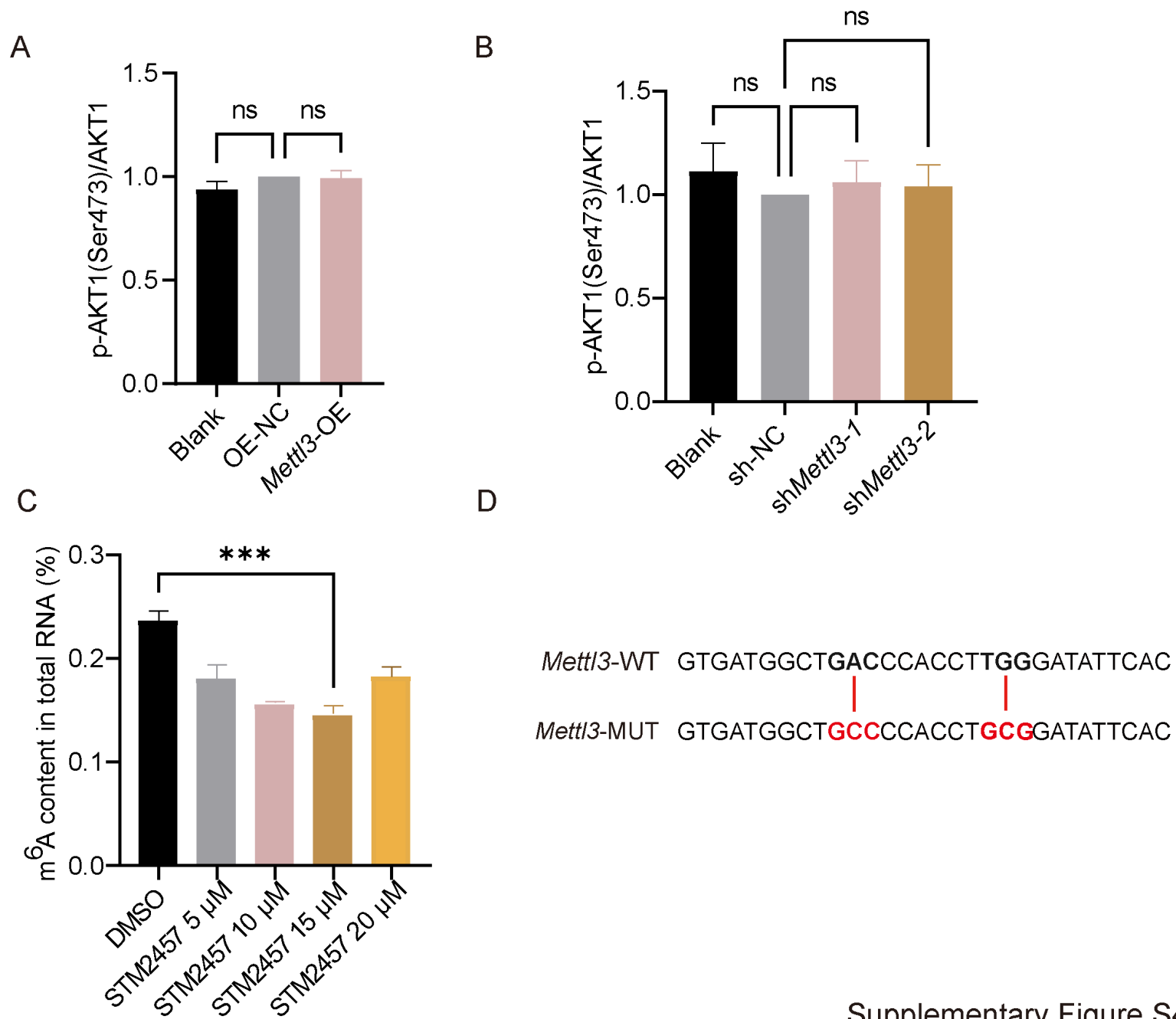

Supplementary Figure S4

Supplement: Supplementary file 4 — Supplementary Figure S4 [file 41419_2023_6325_MOESM4_ESM.pdf]

A

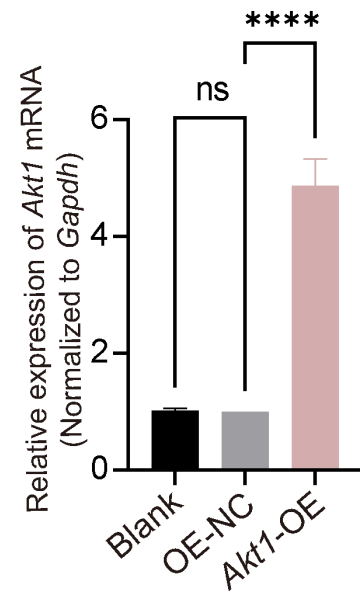

B

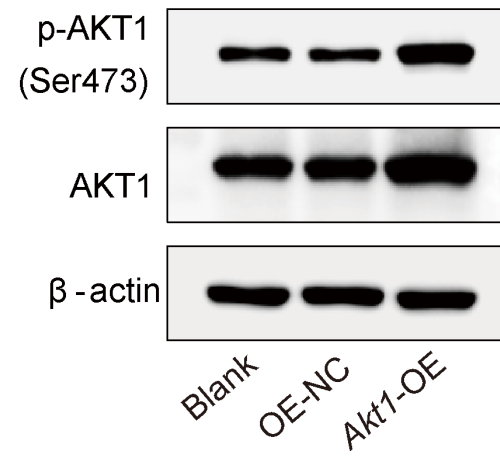

C

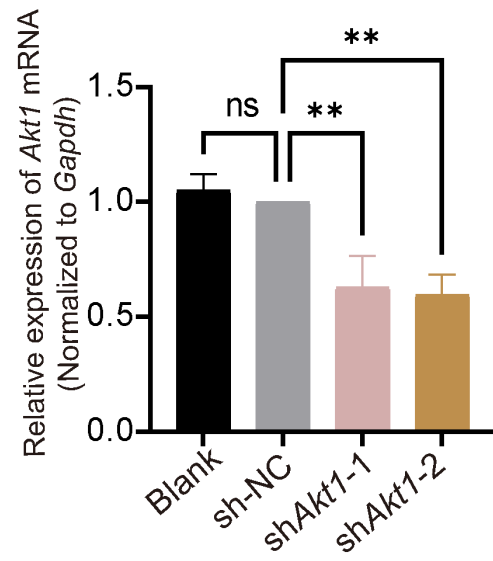

D

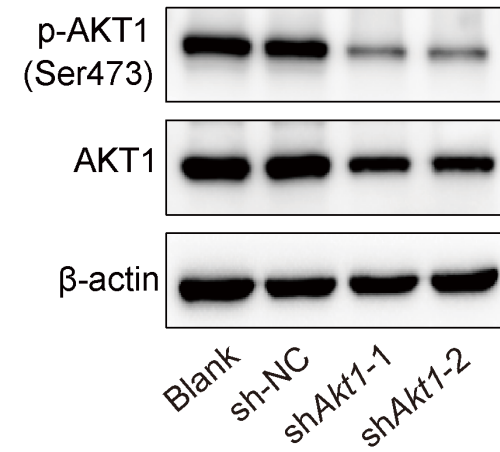

Supplementary Figure S5

Supplement: Supplementary file 5 — Supplementary Figure S5 [file 41419_2023_6325_MOESM5_ESM.pdf]
