# Supplementary material for: Deletion of Mettl3 in mesenchymal stem cells promotes acute myeloid leukemia resistance to chemotherapy
Source: Cell Death Dis. 2023 Dec 5;14(12):796. doi: 10.1038/s41419-023-06325-7 (PMC10698052; doi:10.1038/s41419-023-06325-7)
Supplement: Supplementary file 6 — Supplementary Materials [file 41419_2023_6325_MOESM6_ESM.docx]

**Supplementary Materials**

**Supplementary Figure S1. Generation of conditional *Mettl3* knockout mice. A** Schematic representation of conditional *Mettl3* knockout strategy. The *Mettl3* gene contains the exon 2 to exon 4, which are deleted after Cre-mediated recombination, resulting in translation termination. We crossed *Prrx1-Cre^ERT2^* with *Mettl3^ﬂ/+^*mice to obtain *Prrx1-Cre^ERT2^;Mettl3^ﬂ/+^* mice. By mating *Prrx1-Cre^ERT2^;Mettl3^ﬂ/+^* mice with *Mettl3^fl/fl^* mice, we obtained *Prxx1-Cre^ERT2^;Mettl3^fl/fl^* mice as homozygous conditional *Mettl3* knockout mice. **B** Representative images of PCR genotyping using genomic DNA from tail biopsies.

**Supplementary Figure S2. *Mettl3* deletion in MSCs after AML cell transplantation accelerates AML progression in mice. A** Experimental design. 8-10 weeks-old mice were subjected to irradiation at a dose of 4.5 Gy and *Mettl3* deletion in MSCs was induced one day after AML cell transplantation. **B** Survival of the mice after transplantation (n = 5). **C** The proportion of YFP^+^ cells in peripheral blood was measured weekly by orbital blood after transplantation (n = 5). **D** Representative pictures of livers and spleens obtained from normal mice, *Mettl3^fl/fl^* and *Prxx1-Cre^ERT2^;Mettl3^fl/fl^* AML mice (n = 5). **E** Comparison of the weight of livers and spleens of the *Mettl3^fl/fl^* and *Prxx1-Cre^ERT2^;Mettl3^fl/fl^* AML mice (n = 5). **F** Representative images of H&E staining (top) and the corresponding quantification of AML cells per square millimeter (bottom) of liver, spleen and BM sections from normal mice, *Mettl3^fl/fl^* and *Prxx1-Cre^ERT2^;Mettl3^fl/fl^* AML mice (n = 5). The yellow arrows point to AML cells. Scale Bars: 100 μm, ×100 magniﬁcation. Scale Bars: 20 μm, ×400 magniﬁcation. **G-J** Representative FCM profiles (Red, normal control; Blue, *Mettl3^fl/fl^* or *Prxx1-Cre^ERT2^;Mettl3^fl/fl^* AML mice) and quantification of the percentage of YFP^+^ AML cells (right) in liver (**G**), spleen (**H**), and BM (**I**) and PB (**J**) from *Mettl3^fl/fl^* and *Prxx1-Cre^ERT2^;Mettl3^fl/fl^* mice after transplantation ( n = 5). The data are presented as mean ± SD. Differences were determined using the two tailed unpaired Student’s *t*-test. ***p* < 0.01, ****p* < 0.001, *****p* < 0.0001.

**Supplementary Figure S3. Validate the effects of *Mettl3* gene editing. A** qPCR analysis of *Mettl3* mRNA levels in *Mettl3* overexpressing OP9 cells. **B** Western blot analysis of METTL3 protein levels after *Mettl3* overexpression. **C** Measurement of m^6^A levels in total RNA in *Mettl3* overexpression OP9 cells using EpiQuik m^6^A RNA Methylation Quantiﬁcation Kit. **D** qPCR analysis of *Mettl3* mRNA levels in *Mettl3* knockdown OP9 cells. **E** Western blot analysis of METTL3 protein levels after *Mettl3* knockdown. **F** Measurement of m^6^A levels in total RNA in *Mettl3* knockdown OP9 cells using EpiQuik m^6^A RNA Methylation Quantiﬁcation Kit. Blank, blank control; OE-NC, negative control of overexpression; *Mettl3-*OE, overexpression of mouse *Mettl3*; sh-NC, negative control of knockdown; sh*Mettl3*-1 and sh*Mettl3*-2 independent shRNAs targeting mouse *Mettl3*. Data are presented as mean ± SD. Statistical analysis was performed using one-way ANOVA with Dunnett's multiple comparisons test for multiple groups. ***p* < 0.01, ****p* < 0.001, ns, not signiﬁcant.

**Supplementary Figure S4. OP9 cells were treated with STM2457 or infected with mutant *Mettl3* retroviral particles. A** The relative expression levels of p-AKT1(Ser473)/AKT1 in *Mettl3* overexpression OP9 cells. **B** The relative expression levels of p-AKT1(Ser473)/AKT1 in *Mettl3* knockdown OP9 cells. **C** m^6^A levels in total RNA in OP9 cells treated with different concentrations of STM2457. **D** Exact site of the mutant *Mettl3*. The data are presented as mean ± SD. The one-way ANOVA with Dunnett's multiple comparisons test was performed for multiple groups. Statistical significance was indicated as ****p* < 0.001, ns, not signiﬁcant.

**Supplementary Figure S5. Validation of *Akt1* gene editing. A** qPCR analysis of *Akt1* mRNA levels in *Akt1* overexpressing OP9 cells. **B** Western blot analysis of AKT1 and p-AKT1 (Ser473) protein levels in *Akt1* overexpression OP9 cells. **C** qPCR analysis of *Akt1* mRNA levels after *Akt1* knockdown. **D** Western blot analysis of AKT1 and p-AKT1 (Ser473) protein levels in *Akt1* knockdown OP9 cells. Blank, blank control; OE-NC, negative control of overexpression; *Akt1-*OE, overexpression of mouse *Akt1*; sh-NC, negative control of knockdown; sh*Akt1*-1 and sh*Akt1*-2 independent shRNAs targeting mouse *Akt1*. The data are presented as mean ± SD. The one-way ANOVA with Dunnett's multiple comparisons test was performed for multiple groups. ***p* < 0.01, *****p* < 0.0001, ns, not signiﬁcant.

**Supplementary Table S1.** Primers used for the PCR.

**Supplementary Table S2.** Primers used for the qPCR.
