# Supplementary material for: Deletion of Mettl3 in mesenchymal stem cells promotes acute myeloid leukemia resistance to chemotherapy
Source: Cell Death Dis. 2023 Dec 5;14(12):796. doi: 10.1038/s41419-023-06325-7 (PMC10698052; doi:10.1038/s41419-023-06325-7)
Supplement: Supplementary file 7 — Supplementary Table S1 [file 41419_2023_6325_MOESM7_ESM.docx]

Table S1 Primers used for the PCR

| Gene name | Primers |
| --- | --- |
| *Mettl3*-F(mus) | ATAACCCTGGCTGTCCCG |
| *Mettl3*-R(mus) | TCATTCACATGGCAGCACTT |
| *Cre*-F(mus) | TGTTGGCAAAGGGGTTTTC |
| *Cre*-R(mus) | AGGCAAATTTTGGTGTACGG |
