# Supplementary material for: Deletion of Mettl3 in mesenchymal stem cells promotes acute myeloid leukemia resistance to chemotherapy
Source: Cell Death Dis. 2023 Dec 5;14(12):796. doi: 10.1038/s41419-023-06325-7 (PMC10698052; doi:10.1038/s41419-023-06325-7)
Supplement: Supplementary file 8 — Supplementary Table S2 [file 41419_2023_6325_MOESM8_ESM.docx]

Table S2 Primers used for the qPCR

| Gene name | Primers |
| --- | --- |
| *Mettl3*-F (mus) | CTGGACTGCGATGTGATTGT |
| *Mettl3*-R (mus) | ATCTGGGTCTAGTAGGTGTATCC |
| *Akt1*-F (mus) | AGAAGAGACGATGGACTTCCG |
| *Akt1*-R (mus) | TCAAACTCGTTCATGGTCACAC |
| *Adipoq*-F (mus) | CGTCACTGTTCCCAATGT |
| *Adipoq*-R (mus) | ACCGTGATGTGGTAAGAG |
| *Cebpa*-F (mus) | ACTCCTCCTTTTCCTACCG |
| *Cebpa*-R (mus) | AGGAAGCAGGAATCCTCC |
| *Lpl*-F (mus) | GGGAGTTTGGCTCCAGAGTTT |
| *Lpl*-R (mus) | TGTGTCTTCAGGGGTCCTTAG |
| *Plin1*-F (mus) | CCTGTGGTGAGCGGGACC |
| *Plin1*-R (mus) | GTGGACAGCCGACGGACC |
| *CD36*-F (mus) | GAGCAACTGGTGGATGGTTT |
| *CD36*-R (mus) | GCAGAATCAAGGGAGAGCAC |
| *Pparγ*-F (mus) | AGCCCTTTGGTGACTTTATGG |
| *Pparγ*-R (mus) | CAGCAGGTTGTCTTGGATGT |
