# Supplementary material for: Deletion of Mettl3 in mesenchymal stem cells promotes acute myeloid leukemia resistance to chemotherapy
Source: Cell Death Dis. 2023 Dec 5;14(12):796. doi: 10.1038/s41419-023-06325-7 (PMC10698052; doi:10.1038/s41419-023-06325-7)
Supplement: Supplementary file 9 — Original Data File [file 41419_2023_6325_MOESM9_ESM.pdf]

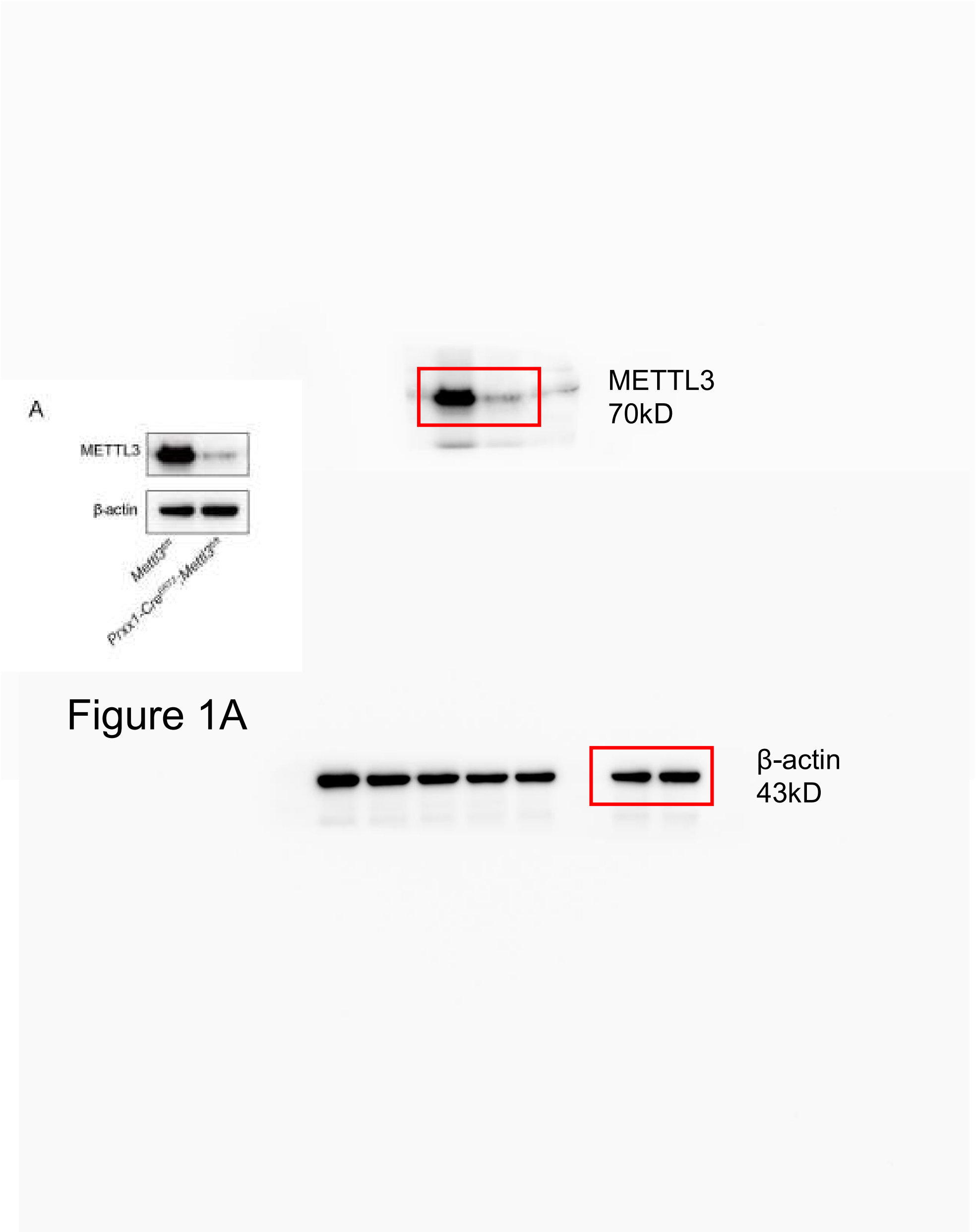

Figure 1A

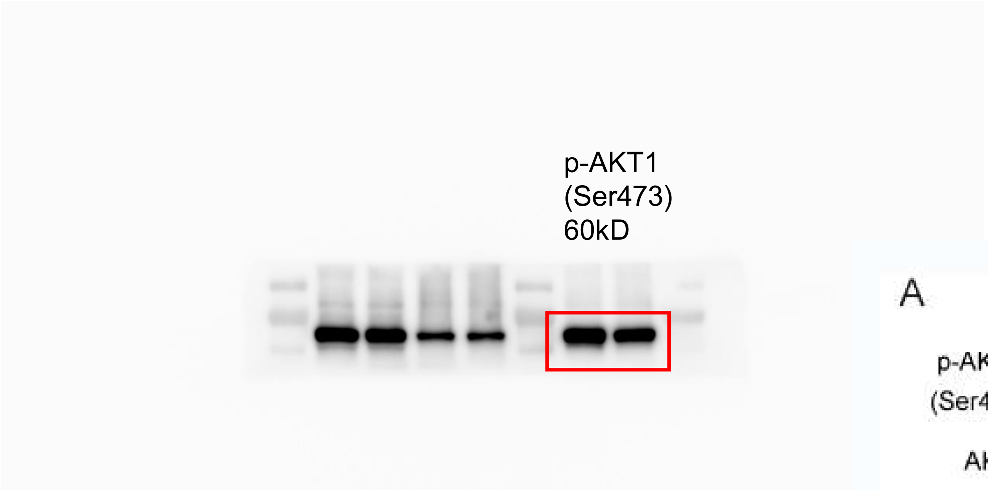
p-AKT1
(Ser473)
60kD

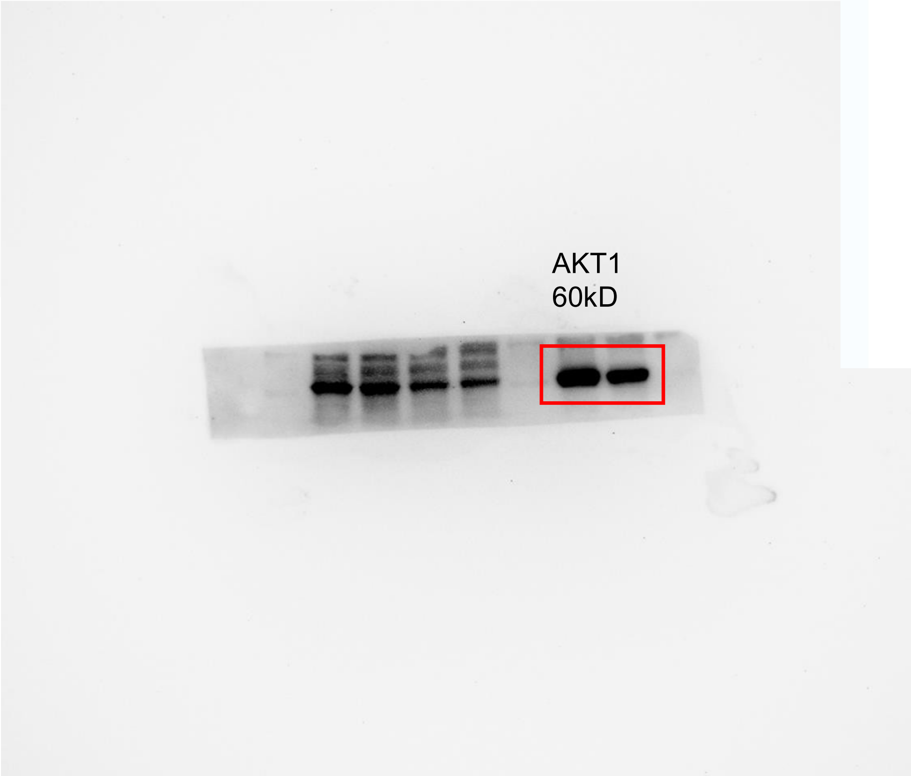
AKT1
60kD

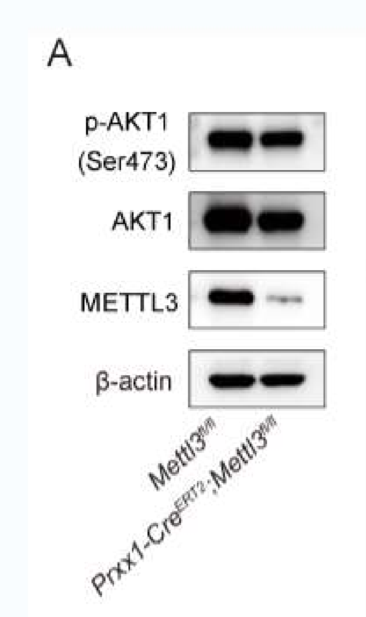
A
p-AKT1
(Ser473)
AKT1
METTL3
β-actin
Mettl3fl/fl
Prrx1-CreERT2;Mettl3fl/fl

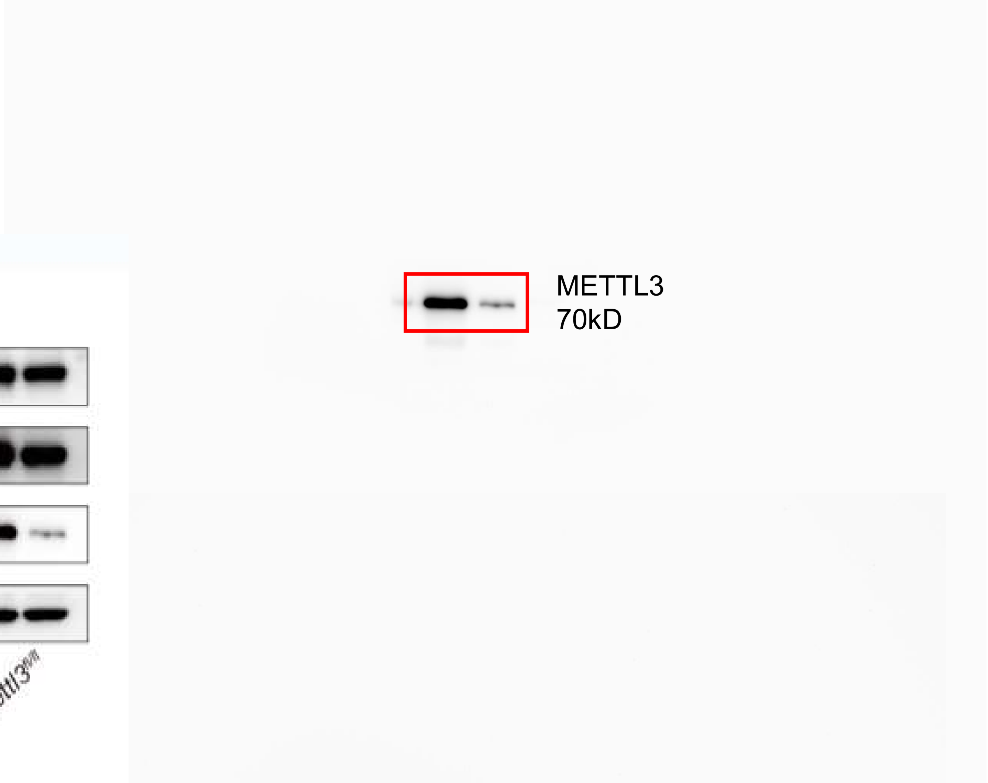
METTL3
70kD

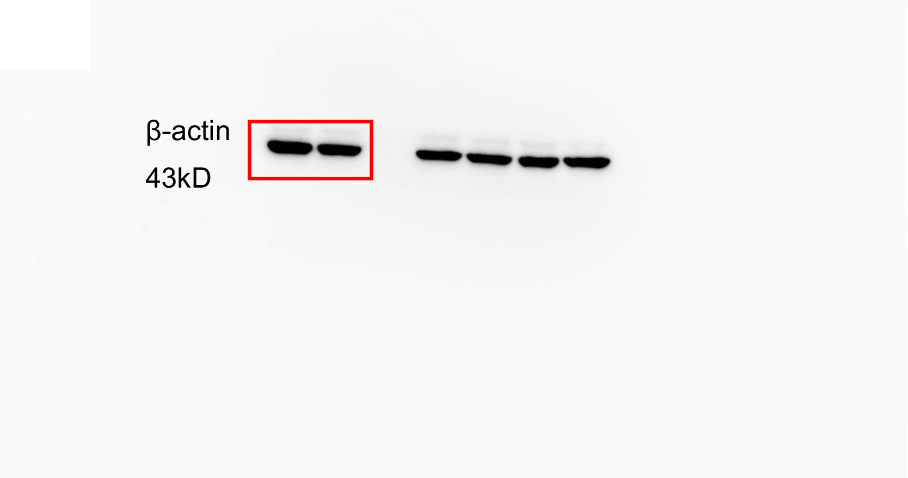
β-actin
43kD

Figure 5A

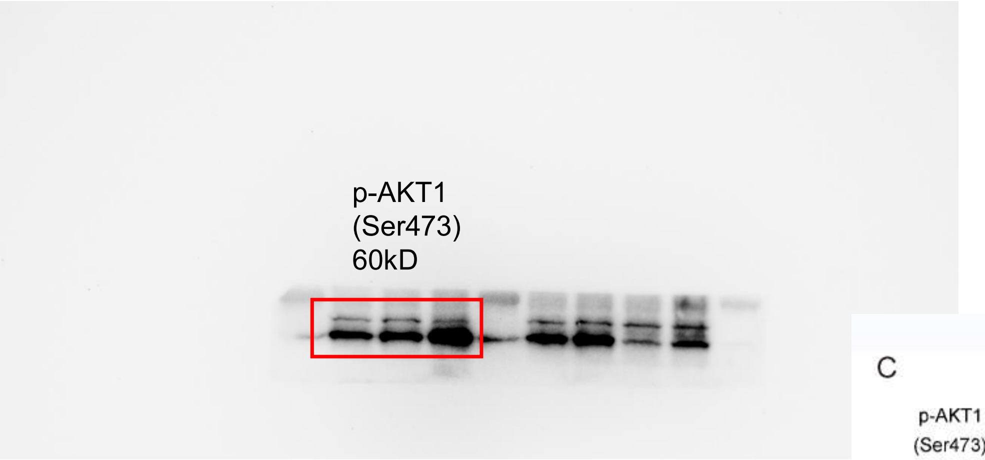
p-AKT1
(Ser473)
60kD

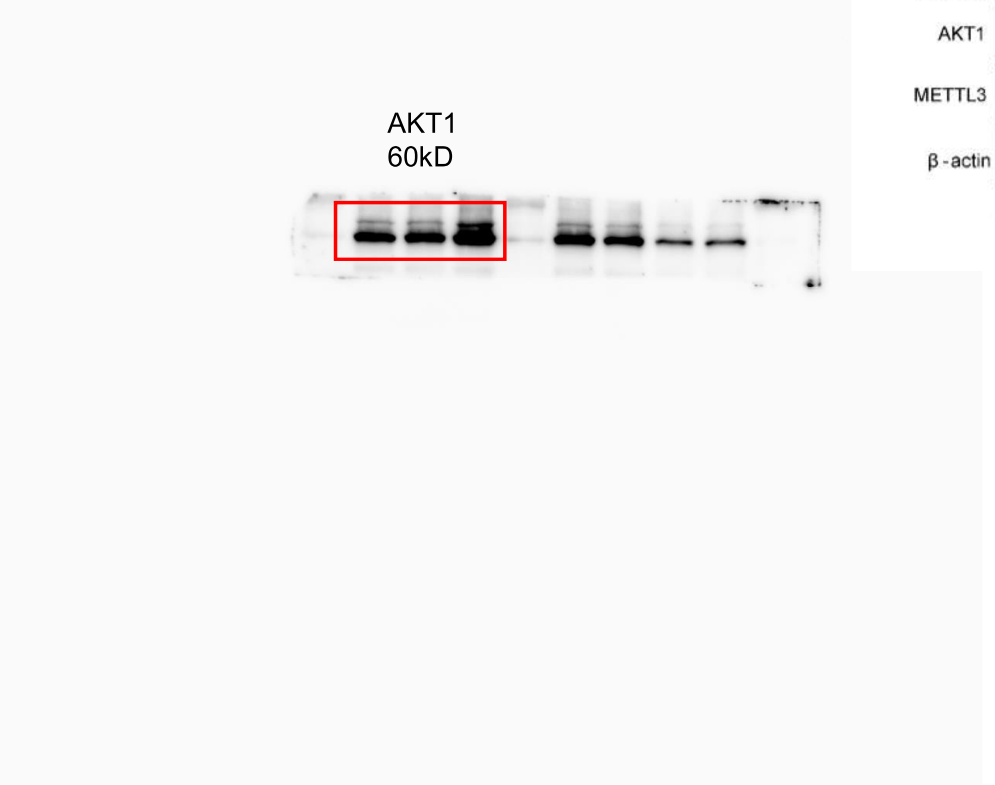
AKT1
60kD

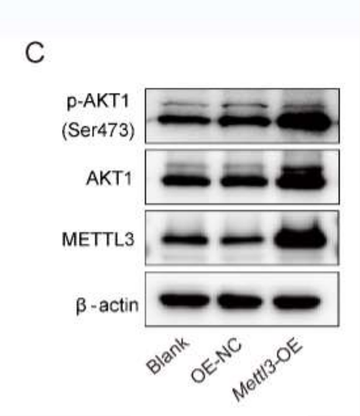
C
p-AKT1
(Ser473)
AKT1
METTL3
β-actin
Blank
OE-NC
Mettl3-OE

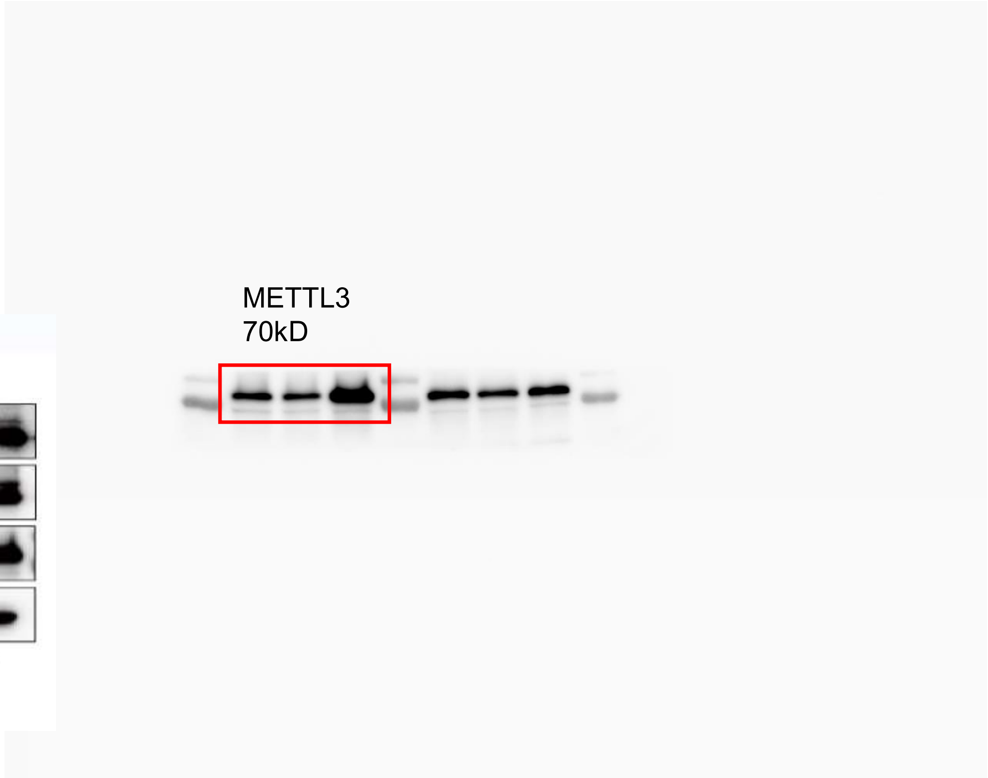
METTL3
70kD

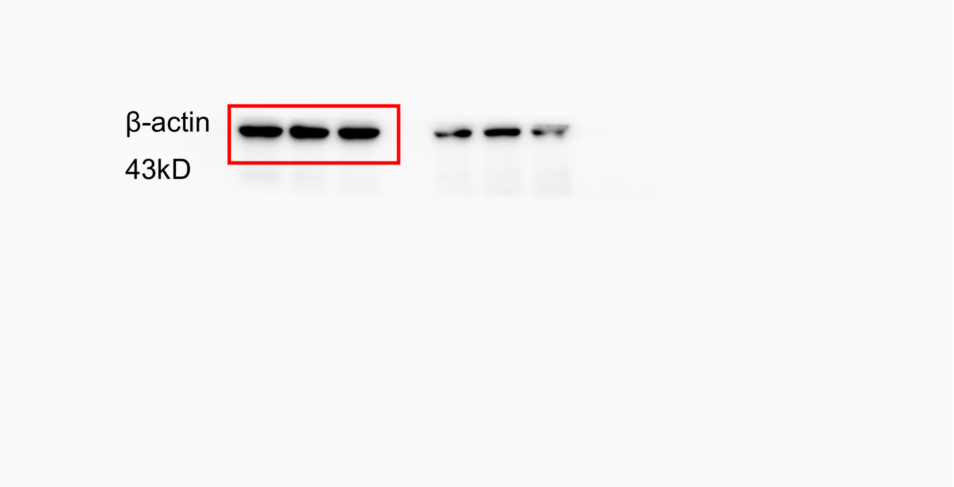
β-actin
43kD

Figure 5C

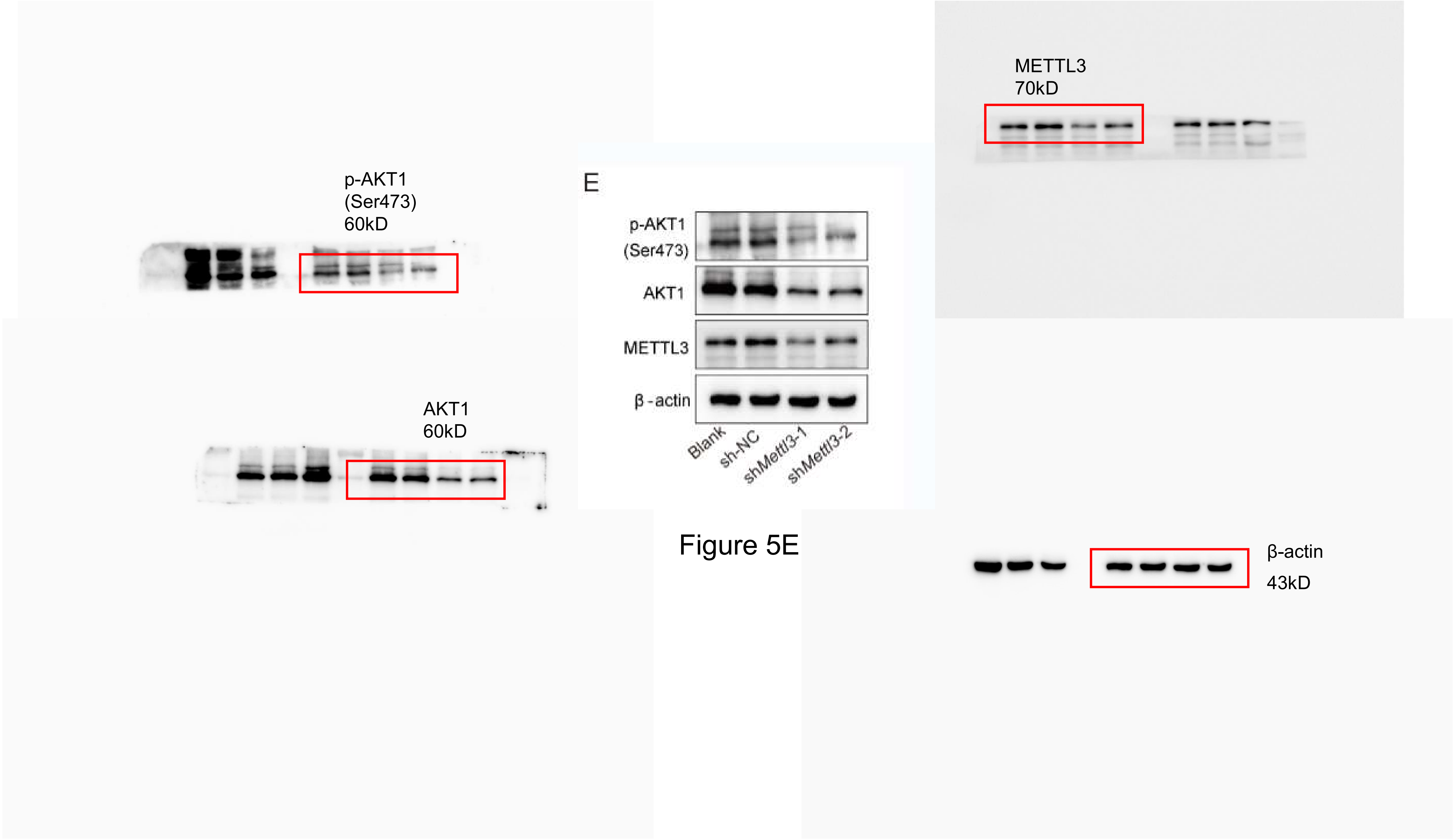
p-AKT1
(Ser473)
60kD
AKT1
60kD
METTL3
70kD
β-actin
43kD
E
p-AKT1
(Ser473)
AKT1
METTL3
β-actin
Blank
sh-NC
shMettl3-1
shMettl3-2

Figure 5E

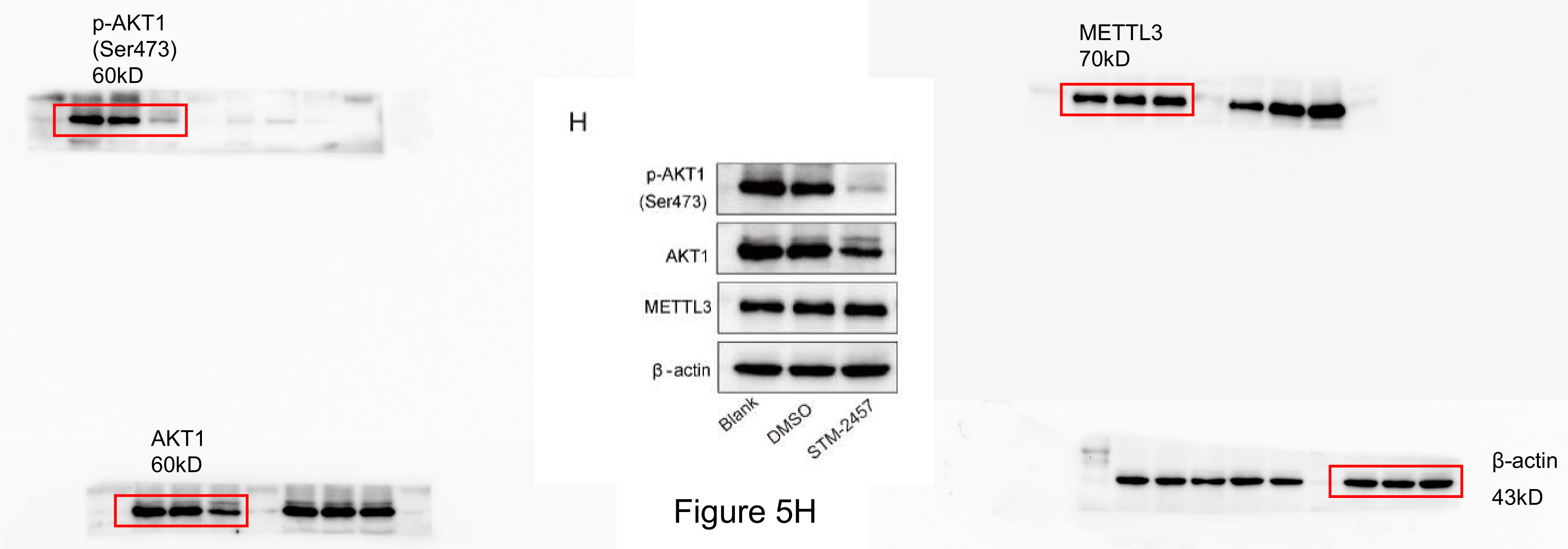
p-AKT1
(Ser473)
60kD
AKT1
60kD
H
p-AKT1
(Ser473)
AKT1
METTL3
β-actin
Blank
DMSO
STM-2457
METTL3
70kD
β-actin
43kD

Figure 5H

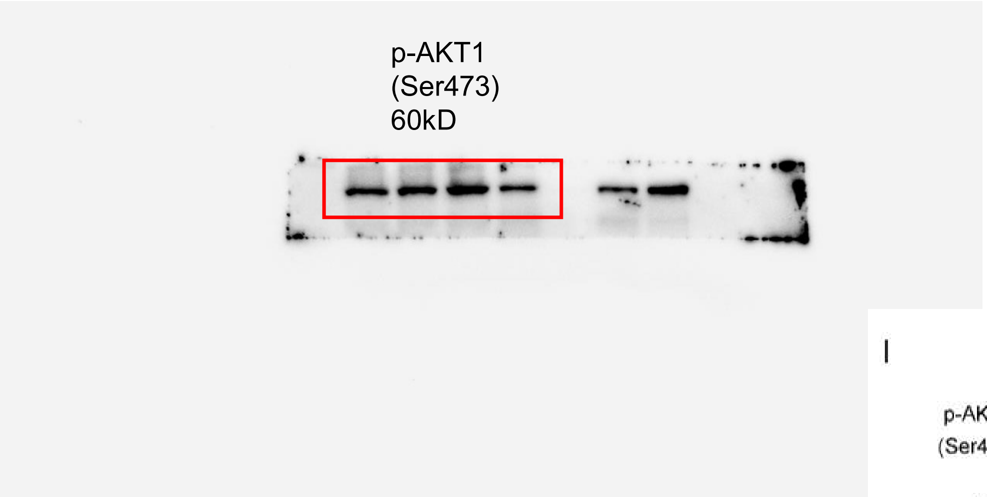
p-AKT1
(Ser473)
60kD

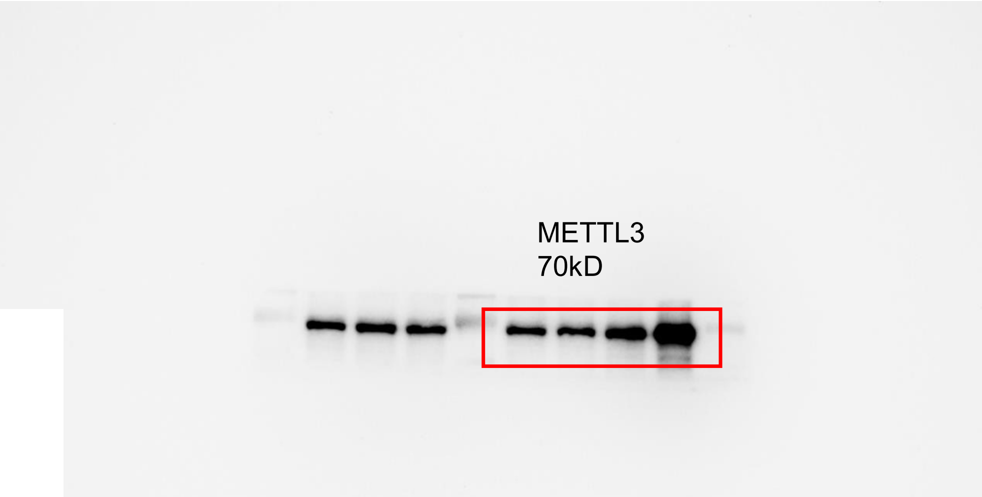
METTL3
70kD

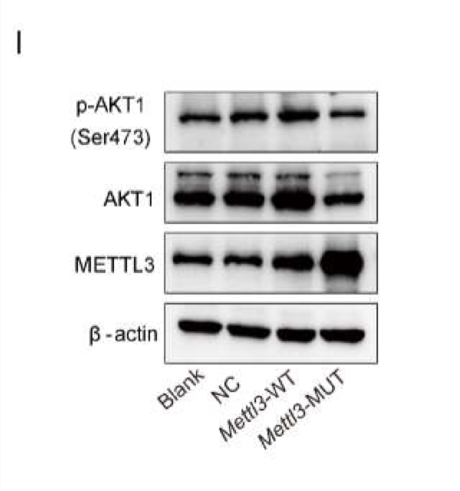
I
p-AKT1
(Ser473)
AKT1
METTL3
β-actin
Blank
NC
Mettl3-WT
Mettl3-MUT

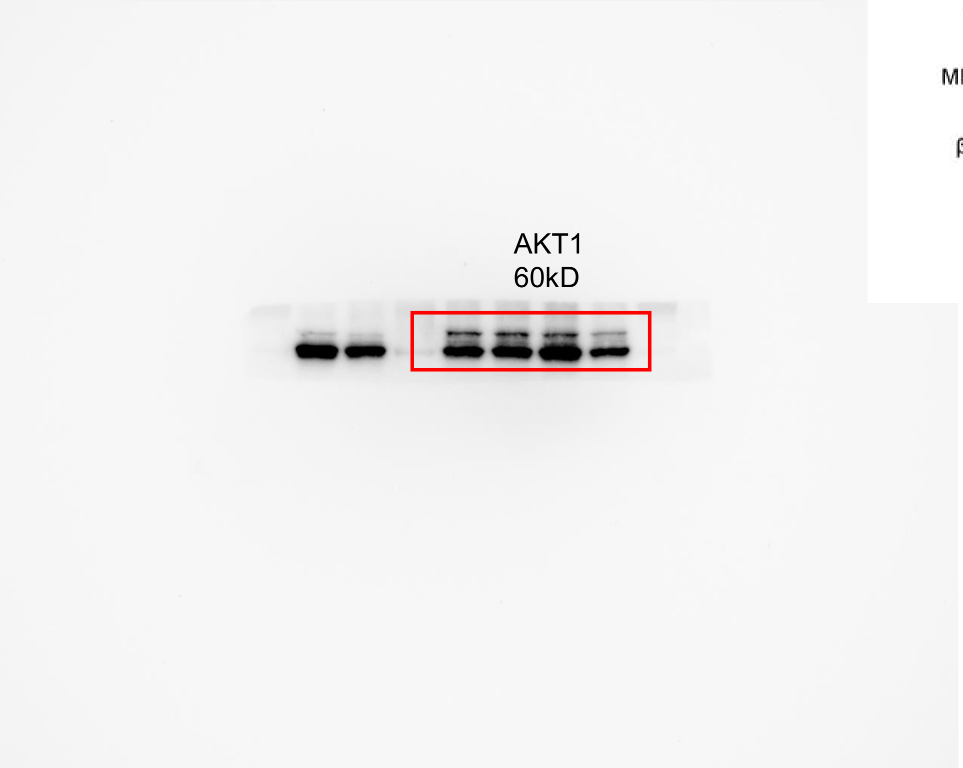
AKT1
60kD

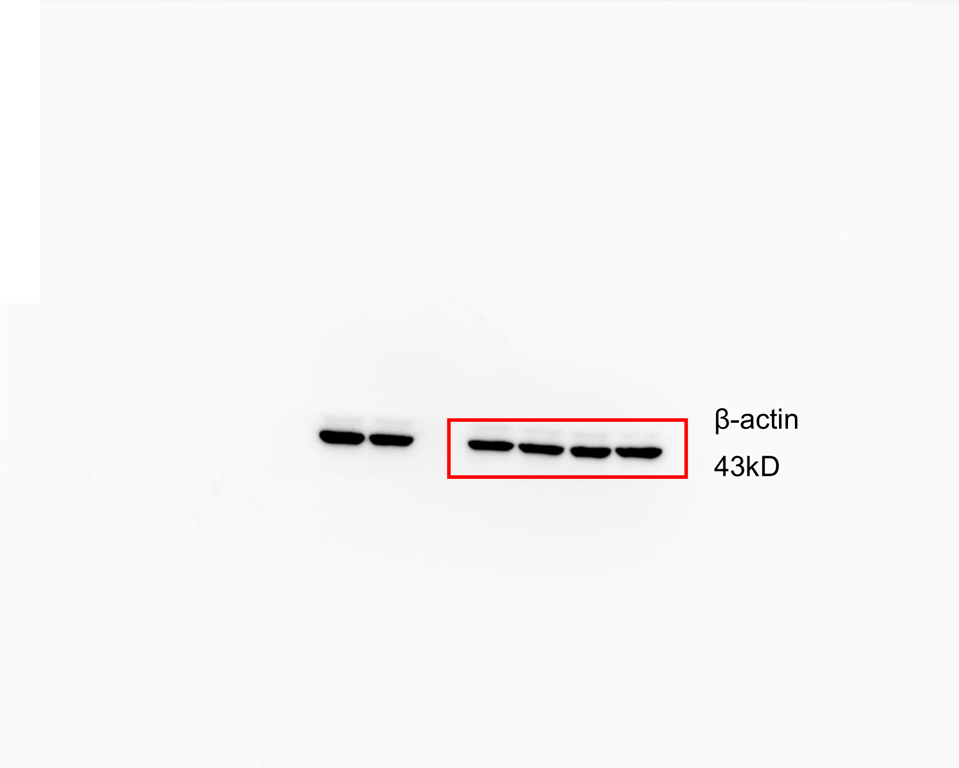
β-actin
43kD

Figure 5I

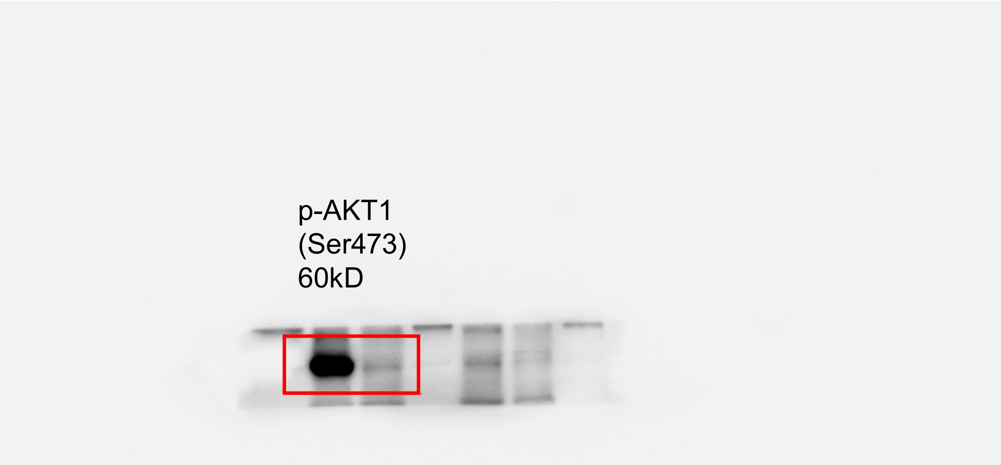
p-AKT1
(Ser473)
60kD

A

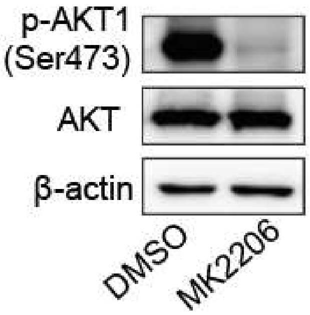
p-AKT1
(Ser473)
AKT
β-actin
DMSO
MK2206

Figure 6A

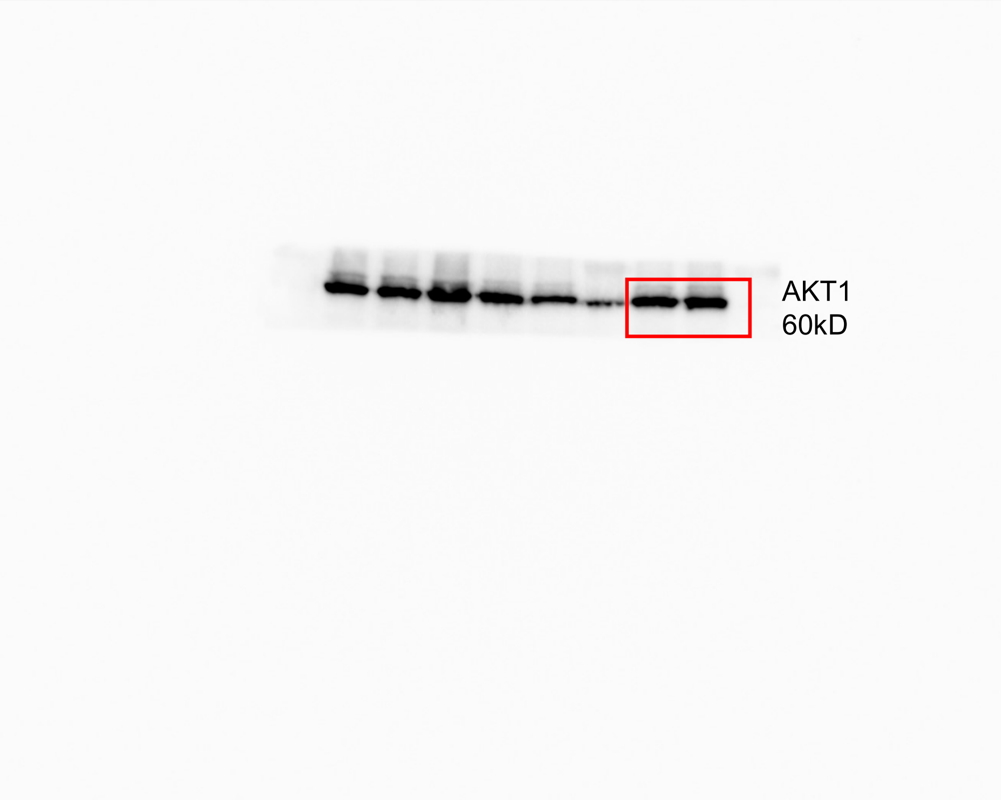
AKT1
60kD

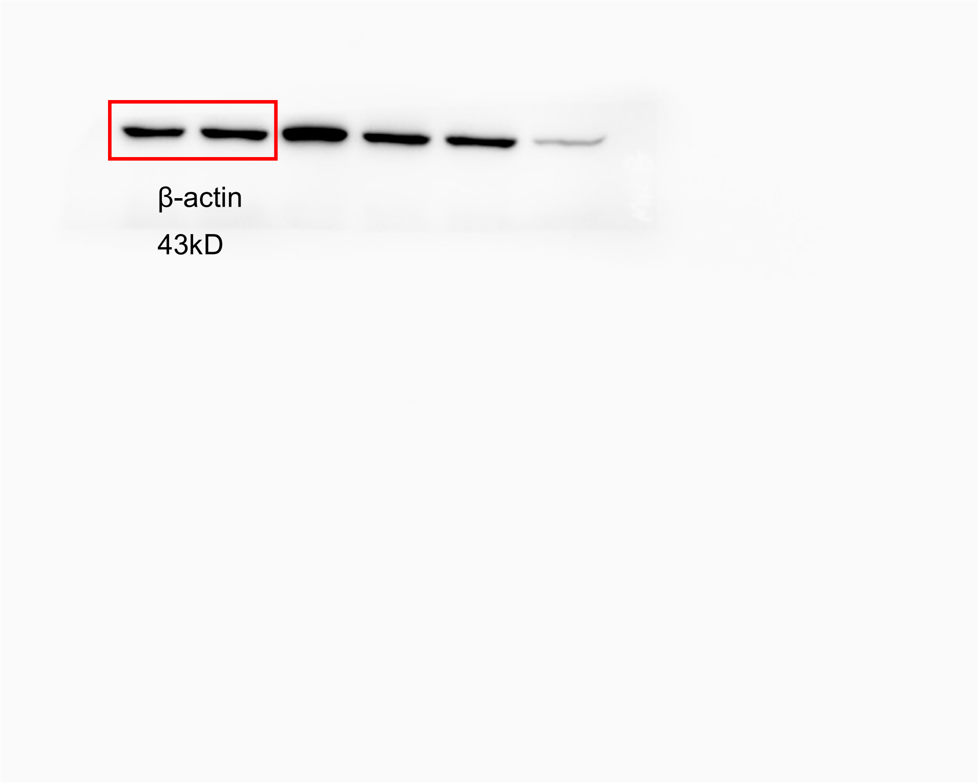
β-actin
43kD

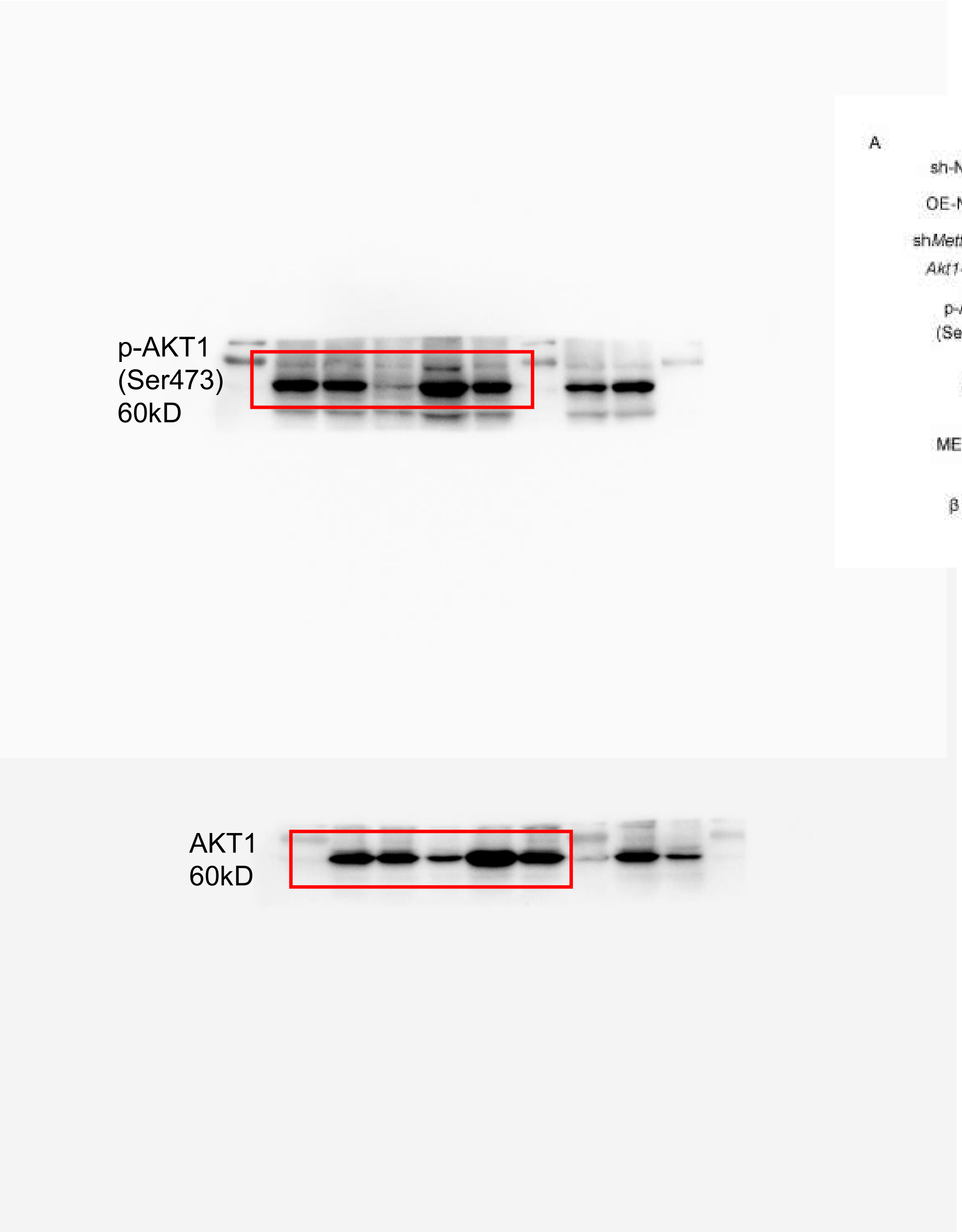
p-AKT1
(Ser473)
60kD
AKT1
60kD

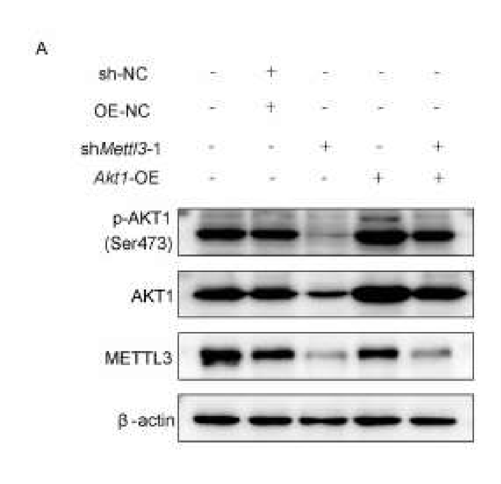
A
sh-NC
OE-NC
shMettl3-1
Akt1-OE
p-AKT1
(Ser473)
AKT1
METTL3
β-actin

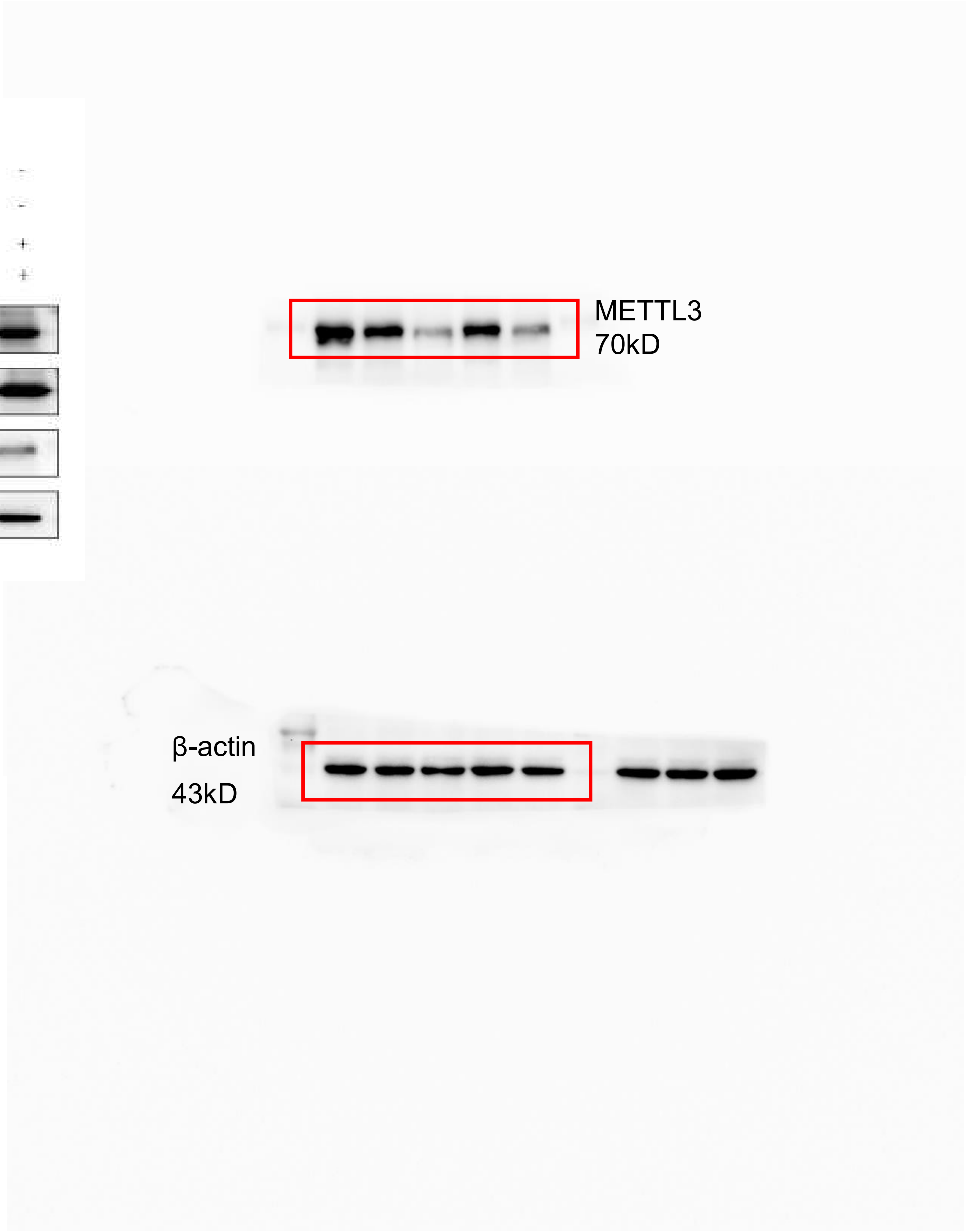
METTL3
70kD
β-actin
43kD

Figure 7A

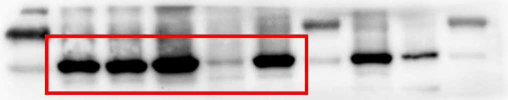

p-AKT1
(Ser473)
60kD

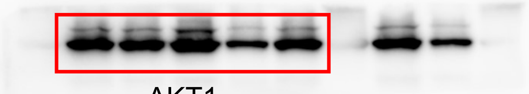

AKT1
60kD

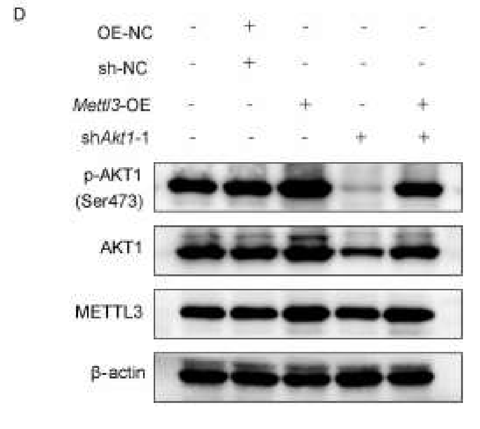

Figure 7D

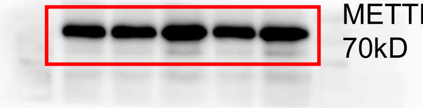

METTL3
70kD

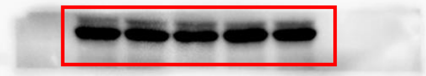

β-actin

43kD

B

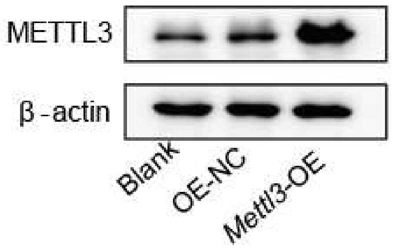

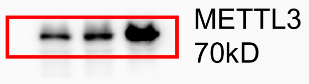

Supplementary Figure S3B

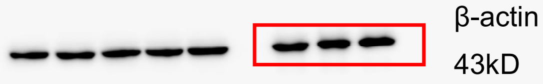

E

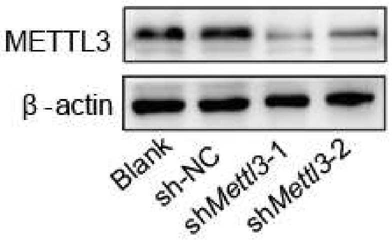

Supplementary Figure S3E

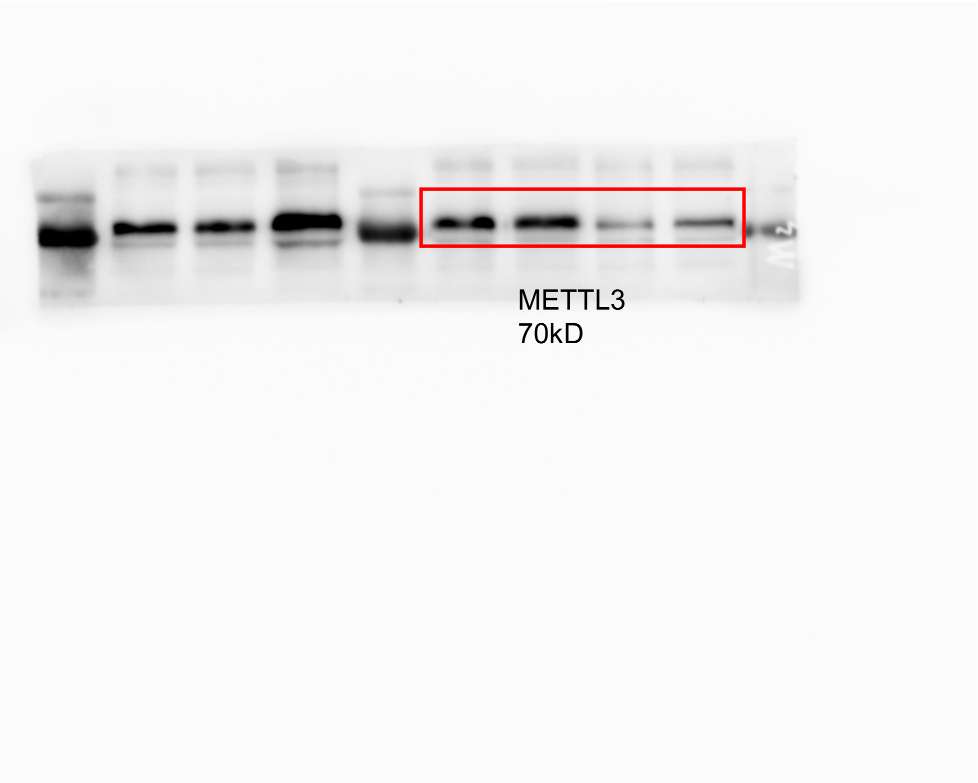

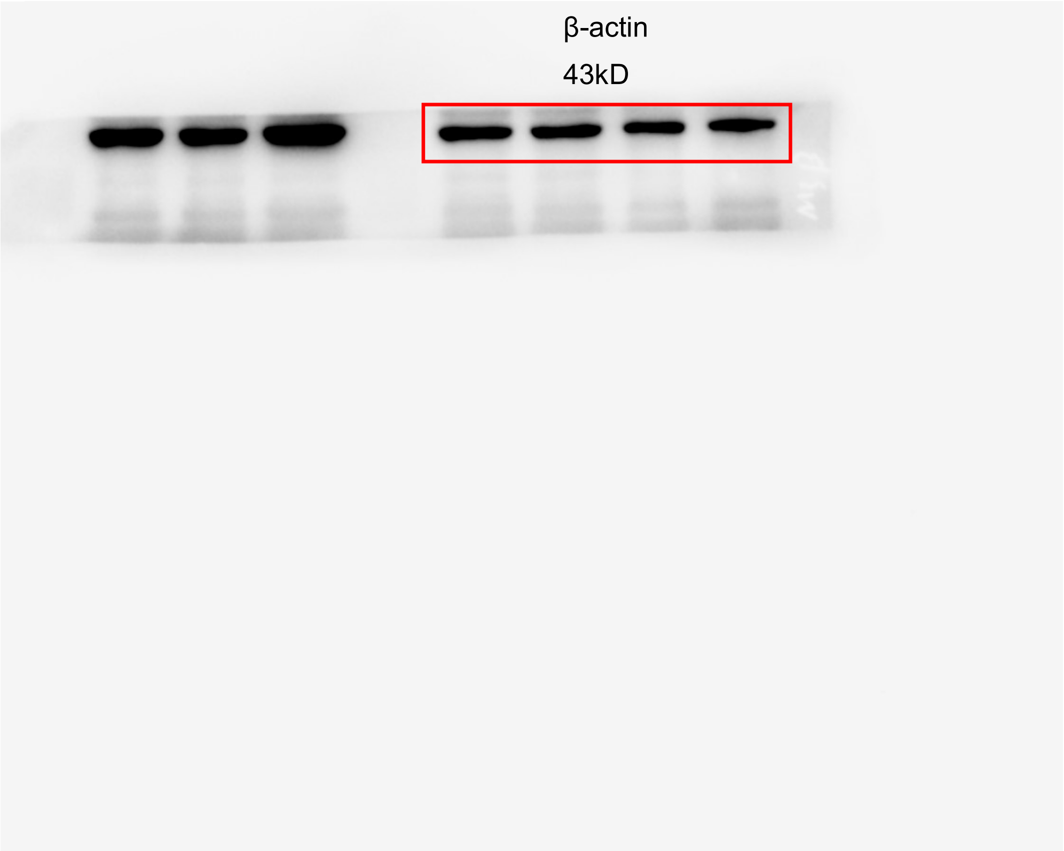

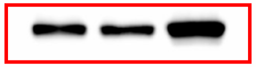

p-AKT1
(Ser473)
60kD

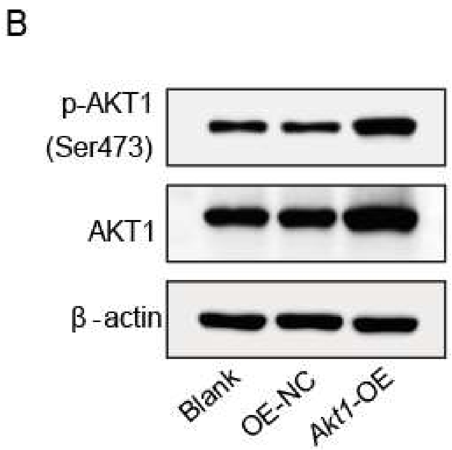

Supplementary
Figure S5B

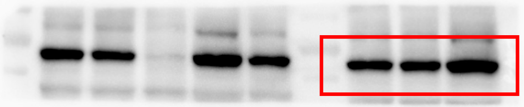

AKT1
60kD

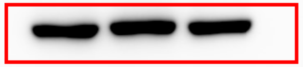

β-actin
43kD

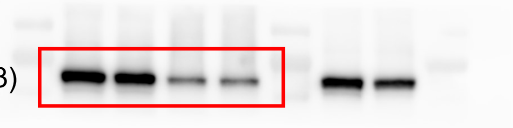

p-AKT1
(Ser473)
60kD

D

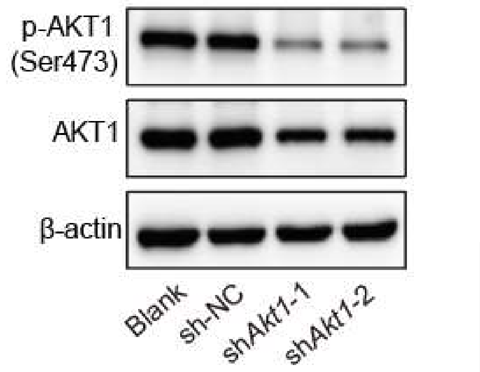

Supplementary
Figure S5D

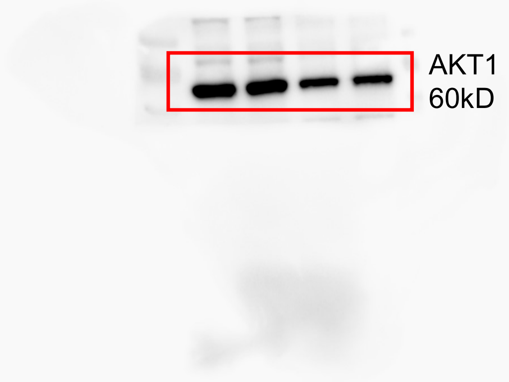

AKT1
60kD

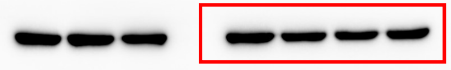

β-actin
43kD
